# Association Between Hormone Therapy and Muscle Mass in Postmenopausal Women: A Systematic Review and Meta-analysis

**DOI:** 10.1001/jamanetworkopen.2019.10154

**Published:** 2019-08-28

**Authors:** Ayesha A. Javed, Alexandra J. Mayhew, Alison K. Shea, Parminder Raina

**Affiliations:** 1 McMaster Institute for Research on Aging, McMaster University, Hamilton, Ontario, Canada; 2 Department of Health Research Methods, Evidence, and Impact, McMaster University, Hamilton, Ontario, Canada; 3 Labarge Centre for Mobility in Aging, McMaster University, Hamilton, Ontario, Canada; 4 Department of Obstetrics and Gynecology, Faculty of Health Sciences, McMaster University, Hamilton, Ontario, Canada

## Abstract

**Importance:**

Hormone therapy (HT) has been suggested for protection against age-related muscle weakness in women. However, the potential for HT-associated health risks necessitates a better understanding of the direction and magnitude of the association between HT and health outcomes, such as lean body mass (LBM).

**Objective:**

To determine whether HT was associated with reduced LBM loss compared with not receiving HT among postmenopausal women aged 50 years and older.

**Data Sources:**

MEDLINE, Embase, AgeLine, CINAHL, and SportDiscus (searched from inception until April 25, 2018).

**Study Selection:**

For this systematic review and meta-analysis, randomized clinical trials including postmenopausal women undergoing HT and control groups of women not receiving HT were selected by 2 reviewers. Studies were included if LBM or fat-free mass were measured as an outcome. Studies with participants from hospitals, long-term care facilities, or with specific diseases were excluded.

**Data Extraction and Synthesis:**

Information regarding study characteristics and outcome measures were extracted by 1 reviewer and verified by another. Risk of bias was evaluated. Quality of evidence was assessed using the Grading of Recommendations Assessment, Development and Evaluation (GRADE) approach. Preferred Reporting Items for Systematic Reviews and Meta-analyses (PRISMA) guidelines were used to abstract data and assess data quality/validity. Data were pooled using a fixed-effects model.

**Main Outcomes and Measures:**

The primary study outcome was the overall absolute change in LBM (measured in kilograms), captured by dual-energy x-ray absorptiometry, dual-photon absorptiometry, or bioelectrical impedance analysis imaging.

**Results:**

Of 8961 studies that met selection criteria, 12 were included, with a total of 4474 recruited participants. Of the participants, mean (SD) age was 59.0 (6.1) years. Data on ethnicity were collected by 2 of the studies. Of the 22 HT intervention arms, 15 used estrogen-progesterone combination HT and 7 used estrogen-only HT. Control participants were women who received no HT at all or who received placebo. The median follow-up duration was 2 years (range, 6 months to 6 years). Seven treatment arms showed a loss of LBM, and 14 were protective. Overall, HT users lost 0.06 kg (95% CI, –0.05 to 0.18) less LBM compared with control participants, but the difference was not statistically significant (*P* = .26). The results were unchanged when stratified based on treatment type and dosage, duration of follow-up, time since menopause, study quality, and type of LBM measurement, with HT users losing between 0.06 kg more to 0.20 kg less LBM compared with control participants for all strata. The quality of evidence based on GRADE was low.

**Conclusions and Relevance:**

This systematic review and meta-analysis did not show a significant beneficial or detrimental association of HT with muscle mass. Although muscle retention in aging women is of crucial importance, these findings suggest that interventions other than HT should be explored.

## Key Points

**Question:**

In postmenopausal women 50 years or older, is estrogen-based hormone therapy associated with reduced loss of lean body mass compared with no hormone therapy?

**Findings:**

In this systematic review and meta-analysis of 12 studies comprising 4474 postmenopausal women, those who received estrogen-based hormone therapy lost less lean body mass compared with women who received no hormone therapy and women who received placebo, but this finding was not statistically significant.

**Meaning:**

The importance of muscle retention in aging women is crucial, but these findings suggest that interventions other than hormone therapy should be explored.

## Teaser

This systematic review and meta-analysis evaluates results of 12 randomized clinical trials, comparing changes in lean body mass in 4474 postmenopausal women who were vs were not receiving estrogen-based hormone therapy.

## Introduction

In 2015, adults 60 years and older composed 12% of the global population. By the year 2050, it is estimated that older adults will compose 22% of the world’s population, numbering approximately 2 billion people.^1^ Women have a longer life expectancy than men but experience more chronic, non–life-threatening illness after the age of 45 years.^2^ One such condition is the age-related decline in muscle mass and strength, called *sarcopenia*. High rates of sarcopenia have been observed in women 60 years and older, and it is hypothesized that the hormone changes occurring at menopause (between 49 and 52 years) may be responsible.^3,4,5^ Individuals with sarcopenia have a greater risk for poor health outcomes, including disability and functional impairments, increased risk of falls, longer hospital stays, and an increased risk of mortality.^6,7,8,9,10,11,12^ Because women live longer than men, women are more likely to experience the negative muscular changes that occur with aging that are strong predictors of mobility and functional impairment.

Accelerated muscle loss, such as that of sarcopenia, has been associated with the menopausal transition and thus linked to declining estrogen levels.^13,14,15,16^ Therefore, hormone therapy (HT) has been suggested as a potential intervention.^17^ Hormone therapy is a method of estrogen supplementation, with or without progesterone, prescribed to manage and treat menopausal symptoms.^18^ However, the exact mechanism between estrogen and muscle mass maintenance has remained elusive.^14,19,20^ Estrogen may be directly involved in muscle metabolism through estrogen receptors found on skeletal muscle,^14,20,21,22,23^ as well as indirectly through the somatotropic axis by altering secretions of growth hormone and insulin growth factor 1.^19,20,24,25^ Also, estrogen plays a role in regulating carbohydrate and lipid metabolism by relieving muscle glycogen and prompting lipid oxidation,^20,26^ which could influence skeletal muscle composition in postmenopausal women.

Despite the potential benefits of HT, data from the Women’s Health Initiative (WHI) study suggested that there may be increased risks associated with HT if started at a later age (ie, after 60 years), including a small increase in risk for stroke and venous thromboembolism.^27^ After the initial publication of the WHI results, a large proportion of women stopped their HT and many health care practitioners were anxious about prescribing HT, despite the relative safety for younger (early menopausal) women. During the window of opportunity in the first 10 years after menopause, HT has multiple health benefits, including relief from menopausal symptoms and reduced risks for coronary heart disease and all-cause mortality.^18,28,29^ However, to our knowledge, there is a lack of consensus among reviews regarding the role of HT in attenuating muscle mass loss. Several reviews have investigated the association between HT use and muscle mass and strength. Some of these reviews have included studies examining resistance training exercise interventions in addition to HT or evaluated muscle performance rather than muscle mass or strength, whereas others have included animal studies to supplement findings in human populations.^30,31,32^ Generally, these reviews have found that HT provides a small, significant benefit in preserving muscle strength (effect size: 0.23; *P* < .05),^31^ and that these benefits may be compounded when HT is used in conjunction with exercise training.^30^ There is also some evidence to suggest that HT may have beneficial effects on muscle mass.^32^ Some observational studies and randomized clinical trials have shown benefits of estrogen therapy on muscle mass in postmenopausal women,^16,33^ while others have not.^13,14,20,34^ However, to our knowledge, there is no systematic review published that has evaluated the independent association between HT use and muscle mass.

The goal of this systematic review and meta-analyses was to determine whether, in postmenopausal women, HT (estrogen only or a combination of estrogen and progesterone) was associated with a reduced loss of muscle mass (measured by lean body mass [LBM] or fat-free mass), compared with not receiving HT, in relation to type and dose of HT, follow-up duration of study, menopausal age of participants, and type of LBM measurement.

## Methods

This study followed the Preferred Reporting Items for Systematic Reviews and Meta-analyses (PRISMA) reporting guideline.^35^ This review was registered in PROSPERO (CRD42016052047), the international prospective register of systematic reviews, on November 30, 2016. Ethics approval was not required for this research.

### Data Sources and Searches

An electronic search strategy was developed to identify human studies investigating the association of HT use in postmenopausal women with LBM. On April 25, 2018, the following electronic databases were searched from inception to April 25, 2018: MEDLINE, Embase (Excerpta Medica Database), AgeLine, CINAHL (Cumulative Index to Nursing and Allied Health), and SportDiscus databases (eTable 1 in the Supplement). The reference lists of all the included studies were also reviewed.

### Inclusion and Exclusion Criteria

Studies were included if participants were community-dwelling postmenopausal women aged 50 years or older who were receiving estrogen-based or estrogen-progesterone–based HT. Studies with participants from hospitals and long-term care facilities, or with specific conditions (breast or other cancer, Turner syndrome, or anorexia) were excluded. No restrictions were placed on the geographic, socioeconomic, or ethnic backgrounds of any of the participants.

Eligible treatments included estrogen-based or estrogen-progesterone–based HT. No other restrictions were placed on HT administration.

Only randomized clinical trials that were complete and published in full were eligible for inclusion in this review. The studies must have conducted primary research in human populations. Animal studies were excluded. We did not place any restrictions on the date of study or publication. Studies were limited to original English-language articles.

Studies were included if LBM or fat-free mass was measured as an outcome. Lean body mass outcomes included measures from body scanning equipment including dual-energy x-ray absorptiometry (DEXA, or DXA), bioelectrical impedance analysis, magnetic resonance imaging, dual-photon absorptiometry, or computed tomography. Studies using muscle circumference or skin calipers for LBM measures were excluded.

### Study Selection

Two independent reviewers (A.A.J. and A.J.M.) screened articles in duplicate at the title and abstract and full-text stages of the review. Screening of studies was conducted using the systematic review software DistillerSR version 2.0 (Evidence Partners).^36^

Any potential conflicts between the reviewers were resolved through discussion. If discrepancies in judgment remain after discussion, a third-party reviewer (P.R.) was consulted to resolve the conflict and provide a final decision.

### Data Extraction

One author (A.A.J.) independently extracted data from the included studies in DistillerSR, and the second author (A.M.) performed verification. Information about the study characteristics, including study date, country of conduct, sample size, age of participants, ethnicity, type of menopause (natural or induced), time since menopause, HT information (type, dose, and duration), type of comparison group, and duration of follow-up, was extracted.

The type of scanning equipment (name, model, coefficient of variation of instrument, timing of measurement) was extracted, as well as LBM values at baseline, all available follow-up, and any data about the amount of change in LBM and *P* values for that change. Any conflicts were resolved through discussion. The third reviewer (P.R.) was consulted if a final decision was required in regard to said disagreements.

### Assessment of Risk of Bias

The 2 independent reviewers (A.A.J. and A.J.M.) assessed the quality of the included studies in duplicate, using the Cochrane Collaboration’s tool for assessing risk of bias in randomized trials.^37^ Any discrepancies between the independent reviewers were resolved by discussion. If conflicts were not adequately resolved through discussion, a third-party reviewer (P.R.) was consulted to resolve said disagreement. The assessment of risk of bias was completed at the study level.

### Grading of Recommendations Assessment, Development and Evaluation

The Grading of Recommendations Assessment, Development and Evaluation (GRADE) approach was used to assess the quality of evidence by outcome.^38,39^ In GRADE, all randomized clinical trials begin with a grade of high and are downgraded based on the presence of risk of bias, inconsistency, indirectness, imprecision, or publication bias. Presence of each factor downgrades study quality by 1 level. The assessment was conducted across all studies, and then further stratified by subgroups. The quality-of-evidence decisions were reviewed and agreed on by both reviewers (A.A.J. and A.J.M.).

The quality of evidence was categorized into 4 levels: very low, low, moderate, and high. Evidence was downgraded for risk of bias if there was evidence of selection, performance, attrition, reporting, or other bias. Evidence was downgraded for inconsistency if the *I*^2^ statistic for the mean difference estimate was greater than 50%, for indirectness if substantial differences existed between the population, intervention, or outcome or if indirect comparisons were used to make inferences about interventions of interest, and imprecision if the optimal information size (400 cases total, with a minimum of 200 cases in the experimental group and 200 in the control group) was not met or if the optimal information size was met but the 95% CI of the mean difference crossed zero. Assessment of publication bias was conducted by a visual assessment of symmetry of the funnel plot. Publication bias was quantitatively assessed using Egger and Begg tests, using SPSS statistical software version 23 (IBM).^40,41^ *P* < .10 was considered evidence of publication bias.

### Data Synthesis and Analysis

Results were synthesized using a quantitative DerSimonian and Laird meta-analysis using Review Manager 5.3 (Cochrane).^42^ A fixed- or random-effects model was used according to heterogeneity, and thus a fixed-effects model was used. An overall absolute change in LBM in kilograms was reported for each treatment arm. Statistical significance was determined at a level of .05 (2-tailed). A summary mean difference in LBM (with 95% CIs) and *I*^2^ statistic were presented for each meta-analysis.

### Subgroup Analyses

Hormone therapy dosage was examined separately within estrogen-only and estrogen-progesterone treatment arms. The various estrogen types used in HT are not equivalent; therefore, all estrogen dosages were standardized using reference values (eTable 2 in the Supplement).^43,44,45^ All values were standardized to those of conjugated equine estrogens, the most commonly used estrogen type across the included studies. Estrogen-only and estrogen-progesterone arms were stratified by an estrogen dose of 0.625 mg or greater (standard dosage or higher) or less than 0.625 mg (low dosage). The proposed mechanism of action for HT on muscle maintenance is through estrogen. Therefore, the estrogen-progesterone treatment arms were not stratified by progesterone dosage.

Follow-up duration subgroups were categorized as longer (>2 years) or shorter (≤2 years). These thresholds were selected because they most evenly dichotomized the study and participant types and captured the range of follow-up duration. Follow-up duration was used as a proxy for total duration of HT because the reported follow-up durations were similar or the same as the duration of HT use across most studies.^46,47,48,49,50,51,52,53^ It is crucial to explore the variability in the duration of estrogen treatment because prolonged exposure to estrogen may be required to pose benefits for LBM preservation. Menopausal age was characterized based on the time since menopausal onset of study participants. Studies with participants with menopausal onset within the past 10 years were included. Generally, it is recommended for postmenopausal women to begin HT use nearer to menopause, specifically within the first 10 years.^54,55^ Time-since-menopause subgroups were also categorized as shorter (<5 years) or longer (≥5 years).

Study quality subgroups were categorized by fair or good quality vs poor quality. Lean body mass measurement type subgroups were categorized as DEXA or other. Dual-energy x-ray absorptiometry is considered the criterion standard of body composition measurement; therefore, all other types were combined and compared against it.^56^

## Results

### Literature Flow

**Figure 1.**
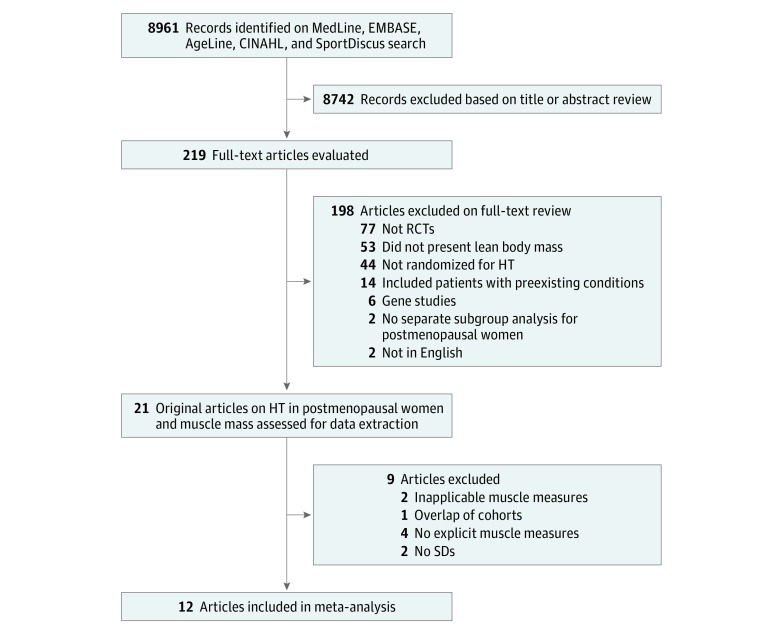
Flow Diagram of the Literature Screening Process HT indicates hormone therapy; RCTs, randomized clinical trials; and SDs, standard deviations.

Among the 21 studies, which included 4474 participants, the mean (SD) age was 59.0 (6.1) years. Data on ethnicity were presented in 2 of the 21 studies. The electronic search of the literature yielded 8961 potentially relevant articles, leaving 219 after the screening of titles and abstracts and 21 articles after full-text screening. Two studies were excluded owing to the method of muscle measurement (skin fold thickness and muscle cross-sectional area), and 1 was excluded because its cohort overlapped with that of another included study. Both of these studies used data from the WHI trials, and the study with the longest duration of follow-up data was used in the analyses.^46,57^ Furthermore, the excluded WHI study did not have the require data for pooling.^57^ Four studies were excluded because they provided qualitative descriptions of changes in LBM and did not have numeric data available for pooling. Two more studies were excluded because they did not have data available for pooling. Twelve studies^46,47,48,49,50,51,52,53,58,59,60,61^ remained and were included in the analysis (Figure 1).

### Study Characteristics

Overall, 6 studies were from the United States and 6 were conducted in Europe. The total number of recruited participants across all 12 applicable studies was 4474, and the median duration of follow-up for studies was 2 years (range, 6 months to 6 years). The age of participants ranged from 45 to 75 years (Table 1).^62^ The full study characteristics are presented in eTables 3 through 5 in the Supplement. The studies had a total of 22 HT treatment arms, 15 of which used estrogen-progesterone combination HT and 7 of which used estrogen-only HT. Treatment duration ranged from 9 to 25 days per month to more than 8 years and varied in dosage. Control participants received either no HT at all or placebo. Eighteen treatment arms consisted of continuous dosage, and 4 used a cyclical dosage regimen. The treatment arms of the included studies depicted varied impacts of the HT, with 7 arms associated with a loss of LBM over the treatment period and 14 arms associated with LBM retention. (Table 2). For the meta-analyses, 21 treatment arms were considered, because the study by Jensen et al^49^ presented results with the 2 HT treatment arms combined. In addition, there were discrepancies between sample sizes in the treatment and control arms for many of the studies. This may indicate fault in randomization or loss to follow-up.

**Table 1.** Study Characteristics^a^

Source	Country	Study Duration	Total Participants Recruited, No./Total in Analysis, No.	Age, Mean (SD), y	Time Since Menopause, Mean (SD), y	HT			Amount of Physical Activity, Mean (SD)
						Type	Dosage, mg/d	Follow-up Period/Duration of HT	
Aloia et al,^60^ 1995	United States	NA	118/77	52.162 (5.7)	2.27 (0.33)	E-P	E: 0.625	E: 25 d/mo	None reported
							P: 10	P: 9 d (days 16-25)	
Bea et al,^46^ 2011	United States	1993-2004	1)^a^ 927/927	1) 63.35 (7.6)	1) 22.21 (8.4)	1) E	0.625	7.7 (1.8) y	E: 10.1 (12.8)^b^
									Control: 9.3 (11.4)^c^
			2) 1014/1014	2) 63.29 (7.2)	2) 13.53 (8.5)	2) E-P	E: 0.625	6.3 (1.5) y	E: 11.4 (14.6)
							P: 2.5		Control: 11.9 (14.6)
Blackman et al,^47^ 2002	United States	1992-1998	28/28	71.5 (5.9)	NA	E-P	E: 100 for 6 mo	E: 6 mo	None reported
							P: 10 for the last 10 d of each 28-d cycle	P: last 10 d of each 28-d cycle for 6 mo (approximately 60-65 d)	
Chen et al,^57^ 2005	United States	1993-2001	835/835; Sensitivity analysis: 511/256 (placebo arm), 511/255 (treatment arm)	63.1 (7.2)	13.8 (8.9)	E-P	E: 0.625	3 y	None reported
							P: 2.5		
Evans et al,^61^ 2001	United States	NA	68/68 (But only 34 in HT and placebo groups combined)	67.7 (5.2)	Mean (SD) age at menopause: 49 (5) y; current mean (SD) age: 67.8 (5) y	E-P	E: 0.625	13 d every third month	None reported
							P: 5		
Haarbo et al,^48^ 1991	Denmark	NA	75/62 (19 HT)	45-55	20.9 (8.4) mo	1) E-P	E: 2	2 y	None reported
							P: 1		
			75/62 (19 HT)		22.4 (9.9) mo	2) E-P	E: 2	2 y	
							P: 75		
Hassager and Christiansen,^58^ 1989	Denmark	1983-1985	133/65	1) 49.91 (2.36)	Inclusion criteria: menopause within the past 0.5-3.0	1) E-P (oral)	E: 2	In a 28-d cycle: E: days 1-11	None reported
							P:	E-P: days 12-21	
								None: days 22-28	
			133/45	2) 50.41 (2.29)		2) E (percutaneous)	E: 0.6	In a 28-d cycle:	
								E: days 1-24, 5 g	
								None: days 25-28	
Jensen et al,^49^ 2003^d^	Denmark	1990-1993	1006/621	50.1 ( 2.8)	0.7 **(**0.6) y	1) E	2	5 y	E: 0.98 (13.02)^e^
						2) E-P	E: 2	In a 28 d cycle:	
							P: 1	E: days 1-12	Control: 1.187 (12.66)
								E-P: days 13-22 E: days 23-28	
Kenny et al,^50^ 2005	United States	NA	167/107	74.3 (6.2)	Mean (SD) age: 74.3 (0.6)	E	0.25 (Ultralow dose)	36 mo	E: Baseline: 120.9 (6.2)^f^
									36 mo: 104.8 (6.6)
									Placebo: baseline: 99.8 (6.1)
									36 mo: 84.7 (6.9)
Pöllänen et al,^51^ 2007	Finland	NA	20/15	53.6 (1.85)	2.8 (3.6)	E-P	E: 2	1 y	None reported
							P: 1		
Sipilä et al,^52^ 2001	Finland	NA	80/52 (30 in HT and placebo groups combined)	50-55	Inclusion criteria: menopause within the past 5 y	E-P	E: 2	1 y	None reported
							P: 1		
Sørensen et al,^59^ 2001	Denmark	NA	16/14	55.5 (2.6)	5.9 (3.9)	E-P	E: 4	In a 28-d cycle; follow-up/duration not specified	None reported
							P: 1	E: 4 mg for 22 d and 1 mg for 6 d; P: 10 d; total: 12 wk	
Thorneycroft et al,^53^ 2007	United States	NA	822/502	51.9 (3.3)	2.2 (0.9)	1) E	1A) E: 0.625	2 y	None reported
				51.5 (4.1)	2.2 (0.9)		1B) E: 0.45	2 y	
				52.0 (3.7)	2.5 (1.0)		1C) E: 0.3	2 y	
				51.5 (3.8)	2.5 (0.9)	2) E-P	1A) E: 0.625	2 y	
							P: 2.5		
				51.1 (3.5)	2.3 (0.9)		1B) E: 0.45	2 y	
							P: 2.5		
				52.3 (3.9)	2.3 (1.0)		1C) E: 0.45	2 y	
							P: 1.5		
				51.3 (3.5)	2.3 (1.0)		1D) E: 0.3	2 y	
							P: 1.5		

Abbreviations: E, estrogen therapy; E-P, estrogen plus progesterone therapy; HT, hormone therapy; NA, not available or not reported; P, progesterone therapy; SE, standard error.

^a^ Numbering refers to treatment group.

^b^ Mean (SD) of baseline weekly energy expenditure (metabolic equivalent values).

^c^ Control groups may have included women receiving placebo or women not receiving HT at all.

^d^ For the meta-analysis, both treatment arms from Jensen et al^49^ have been combined. The study did not provide separate lean body mass measures for treatment arms 1 and 2.

^e^ Mean (SD) of change in amount of exercise (hours per week) across course of study.

^f^ Mean (SE) of total baseline Physical Activity Scale for the Elderly (PASE) score. The PASE is a 5-minute, easily scored survey designed specifically to assess physical activity in epidemiological studies of persons aged 65 years and older. It is self-rated, scores range from 0 to 793, and higher scores indicate greater physical activity.^72^

**Table 2.** Muscle Mass Outcome Measures

Source	Equipment			Timing of Measurements	LBM Measures		*P* Value
	Type	Instrument	Coefficient of Variation, %		Baseline (kg), Mean (SD), % (SD)	Posttreatment Change (kg), Mean (SD), % (SD)	
Aloia et al,^60^ 1995	DPA	Lunar Instruments DP4;^a^ Lunar Radiation, software 1.3	2-3	Baseline, annually	NA	Control: –2.2 (1.6), –5.5 (0.9); treatment: –3.1 (1.6), –7.4% (0.8%)	>.05
Bea et al,^46^ 2011	DEXA	QDR2000, 2000+, or 4500W^b^	NA	Baseline, year 3, year 6	1) Control^c^: 38.82 (5.77), 51.54 (6.31); treatment: 38.49 (5.45), 51.26 (6.52)	1) Control: –0.5 ( 2.45), –0.01 ( 0.06); treatment: –0.44 (2.28), –0.01% (0.06)	*P* = .36 for lean mass and .50 for % lean mass, or .72 for lean mass change and .98 for % lean mass change; between years 1 and 3, the placebo groups lost significantly more than the treatment groups (<.05).
					2) Control: 38.23 (5.42), 53.58 (7.05); treatment: 37.73 (5.17), 53.23 (7.06)	2) Control: –0.4 (2.15), –0.01% (0.05%); treatment: –0.29 (1.99), –0.01% (0.05%)	Between HT and placebo groups, baseline LBM, kg, *P* = .13; baseline % LBM, *P* = .43; change in LBM, kg, *P* = .46; % change in LBM, *P* =.33
Blackman et al,^47^ 2002	DEXA	Lunar model DPX-L^a^	1	Baseline, 6 mo	Control: 35.7 (3.7); treatment: 36.7 (4.1)	Control: 36.1 (4.1); treatment: 37.9 (3.7)	.09 (Change between HT^f^ and placebo)
Evans et al,^61^ 2001	DEXA	QDR-1000/W instrument (version 5.64, enhanced whole body software)^b^	NA	NA	Control: 38.6 (4.0); treatment: 39.1 (5.0)	Control: 0.5 (1.4); treatment: 1.1 (1.9)	NA
Haarbo et al,^48^ 1991	DPA baseline, DEXA follow-up	NA	DPA: 2.1, DEXA: 3.1	DPA at Baseline, DEXA at 2 y	1) Control: 44.7 (3.9); treatment: 43.5 (5.6)	1) Control: 44.0 (3.8); treatment: 43.3 (4.8)	No statistically significant differences between groups, appears to be determined using a 1-way ANOVA
					2) Control: 44.7 (3.9); treatment: 43.3 (3.3)	2) Control: 44.0 (3.8); treatment: 43.9 (4.1)	
Hassager and Christiansen,^58^ 1989	DPA	NA	2.1	Baseline, 2 y	1) Control: 41.5 (6.1); treatment: 39.7 (5.1)	1) Control: 0.33 (2.1); treatment: 0.19 (2.1)	NA
					2) Control: 39.2 (2.8); treatment: 39.9 (3.5)	2) Control: 0.33 (2.1); treatment: 0.81 (1.7)	NA
Jensen et al,^49^ 2003^d^	DEXA	Baseline: QDR whole-body scanners; follow-up: QDR 2000^b^	1.6	Baseline, after 1, 2, and 5 y	NA	Control: –0.02 (2.33); treatment: 0.18 (1.77)	NA
Kenny et al,^50^ 2005	DEXA	DPX-IQ scanner^e^	NA	Baseline, 36 mo	Control: 37.9 (3.7); treatment: 38.1 (3.6)	Control: 37.4 (3.3); treatment: 37.8 (3.4)	Not reported
Pöllänen et al,^51^ 2007	BIA	Spectrum II^f^	NA	Baseline, 12 mo	Control: 49.8 (3.3); treatment: 47.5 (4.0)	Control: 48.4 (2.9); treatment: 48.5 (4.0)	NA
Sipilä et al,^52^ 2001	BIA	Spectrum II^f^	<2	Baseline, 12 mo	Control: 47.4 (5.1 kg); treatment: 45.8 (4.4)	Control: 47.1 (4.2); treatment: 46.9 (4.1)	NA
Sørensen et al,^59^ 2001	DEXA	Norland XR-36 whole body scanner^g^	NA	Baseline, after washout, in week 10	For all participants: 39.0 (4.10)	Control: –0.996 (1.58); treatment:0.347 (0.858)	*t* Test, the difference between change during placebo and change during HT was significant at the .05 level.
Thorneycroft et al,^53^ 2007	DEXA	NA	NA	Baseline, cycles 6, 13, 19, 26 (each cycle is 28 d)	Control: 38.3 (4.0)	Control: 0.19 (1.6), 0.5 (0.42)	None of the changes were statistically different from the placebo group
					1A) Treatment: 38.9 (4.3)	Treatment: –0.12 (1.9), –0.32 (0.5)	
					1B) Treatment: 38.8 ( 4.1)	Treatment: 0.26 (1.6), 0.71 (0.42)	
					1C) Treatment: 37.6 (3.5)	Treatment: –0.04 (1.5), –0.08 (0.42)	
					2A) Treatment: 38.0 (4.0)	Treatment: 0.55 (1.5) 1.47 (0.42)	
					2B) Treatment: 38.7 (4.3)	Treatment: 0.10 (1.5), 0.27 (0.40)	
					2C) Treatment: 39.1 (4.2)	Treatment: 0.13 (1.5), 0.4 (0.4)	
					2D) Treatment: 38.8 (4.4)	Treatment: 0.16 (1.4), 0.56 (0.38)	

Abbreviations: ANOVA, analysis of variance; BIA, bioelectrical impedance analysis; DEXA, dual-energy x-ray absorptiometry; DPA, dual-photon absorptiometry; HT, hormone therapy; LBM, lean body mass; NA, not available or not reported;

^a^ Instruments DP4 is manufactured by Lunar Radiation.

^b^ The QDR2000, 1000/W, 2000+, and 4500W are manufactured by Hologic.

^c^ The numbering of outcome measures in this table is in relation to the estrogen or estrogen-progesterone treatment arms of the studies. The treatment arm characteristics, and HT type and dosage information for each treatment arm are presented in Table 1.

^d^ For the meta-analysis, both treatment arms from Jensen et al^49^ have been combined. The study did not provide separate LBM measures for treatment arms 1 and 2.

^e^ The DPX-IQ scanner is manufactured by GE Medical Systems.

^f^ The Spectrum II is manufactured by RJL Systems.

^g^ The Norland XR-36 whole body scanner is manufactured by Norland Instruments.

### Risk of Bias of Included Studies

Six of the 12 studies (50%) were at high risk of bias, 4 (33%) were unclear, and 2 (17%) were at low risk of bias (Table 2; eTable 6 in the Supplement). Mainly, the studies showed reporting deficiencies, where information was not explicitly stated.

### Meta-analyses

#### Main Effect Analysis

**Figure 2.**
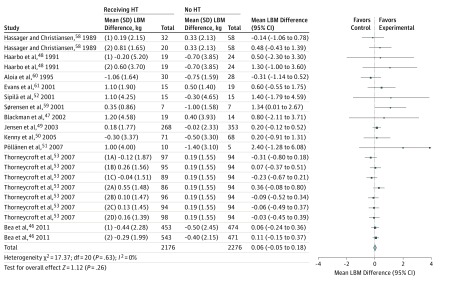
Summary Meta-analysis of the Association Between Hormone Therapy (HT) Intervention and Muscle Mass Outcomes The forest plot of the overall meta-analyses of all included studies presents the mean (95% CI) differences for lean body mass between women receiving HT and women not receiving HT. Size of data marker indicates relative weighting of study.

Across all studies, participants receiving HT lost 0.06 kg less LBM (95% CI, −0.05 to 0.18; *I*^2^ = 0%) compared with those not receiving HT (Figure 2). These findings do not present a statistically significant change in LBM in women receiving HT (*P* = .26).

#### Subgroup Analyses

The studies were stratified and analyzed by the following subgroups: HT type and dosage, duration of follow-up, time since menopause, study quality, and type of LBM measurement. The studies were stratified and analyzed by the following subgroups: HT type and dosage (HT users lost 0.06 kg more to 0.19 kg less LBM than nonusers), duration of follow-up (HT users lost 0.0 to 0.10 kg less LBM than nonusers), time since menopause (HT users lost 0.01 to 0.13 kg less LBM than nonusers), study quality (HT users lost 0.04 to 0.20 kg less LBM than nonusers), and type of LBM measurement (HT users lost 0.06 to 0.07 kg less LBM than nonusers). There were no significant differences in LBM change between women receiving HT and not receiving HT for any group (eTables 7-19 in the Supplement).

#### Publication Bias

A visual inspection of the funnel plot of effect size and precision presents asymmetry, indicating potential publication bias (eFigure in the Supplement). The Egger and Begg tests also suggest publication bias (Egger *P* = .02; Begg *P* = .04).

#### GRADE Assessment

Based on GRADE, the overall quality of evidence was low. In the subgroup analyses, estrogen-progesterone treatment arms with estrogen dosage of 0.625 mg or greater, studies with longer follow-up, shorter and longer time since menopause, poor quality, and other LBM measurement types had low quality of evidence. Subgroups with estrogen-only treatment arms of any dosage, estrogen-progesterone treatment arms with estrogen dosage less than 0.625 mg, shorter follow-up duration, good quality, and DEXA measurement had moderate quality of evidence. Imprecision was a problem for almost all subgroups, and risk of bias was a problem for most. The GRADE assessment of the quality of evidence is presented in eTable 20 in the Supplement.

## Discussion

This systematic review and meta-analyses evaluated 12 randomized clinical trials exploring the role of estrogen-based HT on muscle mass. Overall, HT users lost 0.06 kg (–0.05 to 0.18) less LBM compared with participants not receiving HT. This finding was not statistically significant and is unlikely to be clinically relevant for the average postmenopausal woman. It is reported that women older than 50 years lose approximately 1% of muscle mass annually.^63,64^ At this rate, it would take approximately 66 years for a woman of average height and LBM^65,66^ to become sarcopenic according to the cutoff of 7.4 kg/m^2^ recommended by the European Working Group on Sarcopenia.^67^ Based on the results of this meta-analysis, HT use could increase the amount of sarcopenia-free time to almost 80 years. However, most women would not live long enough to experience these additional sarcopenia-free years. The small potential benefit for maintaining muscle mass in the general population of postmenopausal women likely does not outweigh the potential risks of prolonged HT.^18^ Also, sarcopenia in postmenopausal women is associated most with physical inactivity, reduced protein intake, and oxidative stress occurring at the time of menopause,^68^ but not directly with menopause itself. It is possible this is why HT does not appear to offer any benefit to retaining muscle after menopause, despite the decline in muscle mass during this time.

Hormone therapy could be beneficial to women with a lower muscle mass at baseline; however, to our knowledge, no research in this specific population has been conducted. It has also been hypothesized that HT could be effective in maintaining muscle mass when combined with exercise therapy. However, studies have found that although resistance exercise has a statistically significant association with protection of muscle mass,^69,70^ the combination of exercise and HT does not offer any significant benefit for muscle mass maintenance compared with exercise alone.^14,61,71^

This systematic review and meta-analysis improves on limitations of the existing literature by limiting the scope to HT use and muscle mass. We have provided a comprehensive summary of the available literature on this topic and conducted various subgroup analyses to determine whether the association of HT with LBM users differed based on the estrogen dose, whether progesterone was included, duration of follow-up, time since menopause, method of measuring muscle mass, and study quality. Across all subgroups, women receiving HT lost between 0.06 kg more muscle mass to 0.20 kg less muscle mass compared with the control groups, although none of these subgroup analyses were statistically significant.

Limitations of this review include not being able to explore subgroups such as dosage regimens (cyclical vs continuous), patient characteristics (ie, ethnicity), or amount of physical activity. In this analysis, 4 treatment arms reported using a cyclical dosage regimen and of these, 2 did not report follow-up duration. Most of the included studies did not report ethnicity or amount of exercise. Owing to these factors, we were unable to perform these subgroup analyses. Although we believe it is unlikely for future studies to find an association between HT use and attenuation of LBM loss in postmenopausal women, studies could improve on the current literature by providing data on these subgroups as well as using longer follow-up. Studies may also consider focusing on women with lower LBM at baseline to evaluate the potential benefit in a higher-risk population. In addition, our analysis exploring the window of opportunity for HT less than 10 years after menopausal onset was limited because the studies were not designed to include women in very early menopause. Our follow-up duration subgroup analysis was also limited because we used follow-up duration as a proxy measure for total duration of HT use, as some studies did not explicitly report the latter.^49,58,60,61^

Further work is also required to determine whether HT is beneficial to muscle strength or function. Muscle strength is more important to health outcomes than muscle mass^72^; however, we are not aware of any biological link between HT and muscle strength that would not be mediated through muscle mass, hence the reason this analysis focused on the latter. A previous systematic review and meta-analysis of 23 human studies has shown small, significant benefits of HT in preserving skeletal muscle strength, translating to approximately 5% greater strength in HT users compared with control participants.^31^ However, the type, dosage, and duration of HT among these studies were not consistent and varied greatly from study to study. Therefore, further work in this area is required.

### Limitations

This study had several limitations. Many of the studies used in our review were considered to have a high risk of bias and the overall quality of evidence was low based on GRADE. The quality of evidence was commonly downgraded owing to study risk of bias, publication bias, or imprecision. Imprecision was present when studies did not meet the optimal information size or if the 95% CI of the mean difference crossed zero. However, this definition of imprecision is difficult to interpret if we assume that there is a null association and that the CI should include zero. In addition, the presence of publication bias shows that smaller studies with larger, significant effects are more likely to be published. In a meta-analysis, this can skew results in favor of the treatment, whether or not a true effect exists. Despite the literature’s limitations, the results of this review remained consistent across subgroups, indicating that the overall body of literature has not shown a meaningful association between HT and muscle mass. Of the 12 included studies, 1 had a statistically significant result; however, it was severely limited by a sample size of 14 participants.

## Conclusions

This systematic review and meta-analysis of 12 randomized clinical trials exploring muscle mass retention in postmenopausal women did not show a significant beneficial or detrimental association of HT with muscle mass. Pooling data across all studies, participants using HT lost 0.06 kg (95% CI, –0.05 to 0.18 kg; *P* = .26) less LBM compared with the control participants, and significant between study heterogeneity remained. Individual study effects ranged from –0.06 kg (–0.30 to 0.19 kg) to 0.20 kg (–0.08 to 0.48 kg). Findings from subgroup analyses by follow-up duration, time since menopause, study quality, estrogen dosage, and LBM measurement type were not statistically significant. Despite the limitations of the literature, this study highlights the consistently null results in studies investigating HT and retention of muscle mass. The importance of muscle retention in aging women is crucial, but these findings suggest that interventions other than HT should be explored.

## Notes

Supplement. **eTable 1.** Electronic Search Strategies for Databases MEDLINE, Embase, AgeLine, CINAHL, and SportDiscus **eTable 2.** Estrogen Dose Equivalence Calculations **eTable 3.** Study Characteristics (Part 1) **eTable 4.** Study Characteristics (Part 2) **eTable 5.** Study Characteristics (Part 3) **eTable 6.** Risk of Bias Assessment **eTable 7.** Summary Meta-analysis of the Association Between Less Than 0.625 mg Estrogen-Only Treatment and Muscle Mass Outcomes **eTable 8.** Summary Meta-analysis of the Association Between 0.625 mg or More Estrogen-Only Treatment and Muscle Mass Outcomes **eTable 9.** Summary Meta-analysis of the Association Between Less than 0.625 mg Estrogen + Any Dose Progesterone Treatment and Muscle Mass Outcomes **eTable 10.** Summary Meta-analysis of the Association Between 0.625 mg or More Estrogen + Any Dose Progesterone Treatment and Muscle Mass Outcomes **eTable 11.** Summary Meta-analysis of the Association Between Shorter Follow-up Lengths and Muscle Mass Outcomes **eTable 12.** Summary Meta-analysis of the Association Between Longer Follow-up Lengths and Muscle Mass Outcomes **eTable 13.** Summary Meta-analysis of Studies With <10 Years of Time Since Menopause **eTable 14.** Summary Meta-analysis of the Association Between Shorter Times Since Menopause and Muscle Mass Outcomes **eTable 15.** Summary Meta-analysis of the Association Between Longer Times Since Menopause and Muscle Mass Outcomes **eTable 16.** Summary Meta-analysis of the Association Between Fair/Good Study Quality and Muscle Mass Outcomes **eTable 17.** Summary Meta-analysis of the Association Between Poor Study Quality and Muscle Mass Outcomes **eTable 18.** Summary Meta-analysis of the Association Between DEXA Measurement and Muscle Mass Outcomes **eTable 19.** Summary Meta-analysis of the Association Between Other Measurement and Muscle Mass Outcomes **eTable 20.** GRADE Assessment **eFigure.** Funnel Plot for Assessment of Publication Bias
