# Supplementary material for: Association Between Hormone Therapy and Muscle Mass in Postmenopausal Women: A Systematic Review and Meta-analysis
Source: JAMA Netw Open. 2019 Aug 28;2(8):e1910154. doi: 10.1001/jamanetworkopen.2019.10154 (PMC6716293; doi:10.1001/jamanetworkopen.2019.10154)
Supplement: Supplement. — eTable 1. Electronic Search Strategies for Databases MEDLINE, Embase, AgeLine, CINAHL, and SportDiscus eTable 2. Estrogen Dose Equivalence Calculations eTable 3. Study Characteristics (Part 1) eTable 4. Study Characteristics (Part 2) eTable 5. Study Characteristics (Part 3) eTable 6. Risk of Bias Assessment eTable 7. Summary Meta-analysis of the Association Between Less Than 0.625 mg Estrogen-Only Treatment and Muscle Mass Outcomes eTable 8. Summary Meta-analysis of the Association Between 0.625 mg or More Estrogen-Only Treatment and Muscle Mass Outcomes eTable 9. Summary Meta-analysis of the Association Between Less than 0.625 mg Estrogen + Any Dose Progesterone Treatment and Muscle Mass Outcomes eTable 10. Summary Meta-analysis of the Association Between 0.625 mg or More Estrogen + Any Dose Progesterone Treatment and Muscle Mass Outcomes eTable 11. Summary Meta-analysis of the Association Between Shorter Follow-up Lengths and Muscle Mass Outcomes eTable 12. Summary Meta-analysis of the Association Between Longer Follow-up Lengths and Muscle Mass Outcomes eTable 13. Summary Meta-analysis of Studies With <10 Years of Time Since Menopause eTable 14. Summary Meta-analysis of the Association Between Shorter Times Since Menopause and Muscle Mass Outcomes eTable 15. Summary Meta-analysis of the Association Between Longer Times Since Menopause and Muscle Mass Outcomes eTable 16. Summary Meta-analysis of the Association Between Fair/Good Study Quality and Muscle Mass Outcomes eTable 17. Summary Meta-analysis of the Association Between Poor Study Quality and Muscle Mass Outcomes eTable 18. Summary Meta-analysis of the Association Between DEXA Measurement and Muscle Mass Outcomes eTable 19. Summary Meta-analysis of the Association Between Other Measurement and Muscle Mass Outcomes eTable 20. GRADE Assessment eFigure. Funnel Plot for Assessment of Publication Bias [file jamanetwopen-2-e1910154-s001.pdf]

Supplementary Online Content

Javed AA, Mayhew AJ, Shea AK, Raina P. Association between hormone therapy and muscle mass in postmenopausal women: a systematic review and meta-analysis. *JAMA Netw Open*. 2019;2(8):e1910154. doi:10.1001/jamanetworkopen.2019.10154

eTable 1. Electronic Search Strategies for Databases MEDLINE, Embase, AgeLine, CINAHL, and SportDiscus
eTable 2. Estrogen Dose Equivalence Calculations
eTable 3. Study Characteristics (Part 1)
eTable 4. Study Characteristics (Part 2)
eTable 5. Study Characteristics (Part 3)
eTable 6. Risk of Bias Assessment
eTable 7. Summary Meta-analysis of the Association Between Less Than 0.625 mg Estrogen-Only Treatment and Muscle Mass Outcomes
eTable 8. Summary Meta-analysis of the Association Between 0.625 mg or More Estrogen-Only Treatment and Muscle Mass Outcomes
eTable 9. Summary Meta-analysis of the Association Between Less than 0.625 mg Estrogen + Any Dose Progesterone Treatment and Muscle Mass Outcomes
eTable 10. Summary Meta-analysis of the Association Between 0.625 mg or More Estrogen + Any Dose Progesterone Treatment and Muscle Mass Outcomes
eTable 11. Summary Meta-analysis of the Association Between Shorter Follow-up Lengths and Muscle Mass Outcomes
eTable 12. Summary Meta-analysis of the Association Between Longer Follow-up Lengths and Muscle Mass Outcomes
eTable 13. Summary Meta-analysis of Studies With <10 Years of Time Since Menopause
eTable 14. Summary Meta-analysis of the Association Between Shorter Times Since Menopause and Muscle Mass Outcomes
eTable 15. Summary Meta-analysis of the Association Between Longer Times Since Menopause and Muscle Mass Outcomes
eTable 16. Summary Meta-analysis of the Association Between Fair/Good Study Quality and Muscle Mass Outcomes
eTable 17. Summary Meta-analysis of the Association Between Poor Study Quality and Muscle Mass Outcomes
eTable 18. Summary Meta-analysis of the Association Between DEXA Measurement and Muscle Mass Outcomes
eTable 19. Summary Meta-analysis of the Association Between Other Measurement and Muscle Mass Outcomes
eTable 20. GRADE Assessment
eFigure. Funnel Plot for Assessment of Publication Bias

This supplementary material has been provided by the authors to give readers additional information about their work.

©Javed AA et al. *JAMA Network Open*.

eTable 1. Electronic Search Strategies for Databases MEDLINE, Embase, AgeLine, CINAHL, and SportDiscus

Database	Search strategy		
	**#**	**Search term**	**Results (n)**
**MEDLINE**	1	exp Hormone Replacement Therapy/ or hormon* replacement therap*.mp.	30785
	2	(hormon* adj2 therap*).mp.	45612
	3	(hormon* adj2 replac*).mp.	23721
	4	HRT.mp.	9782
	5	(hormon* adj2 supplement*).mp.	1344
	6	exp Estrogen Replacement Therapy/ or estrogen replacement therap*.mp.	17068
	7	(estrogen adj2 therap*).mp.	20357
	8	estrogen.mp. or exp Estrogens/	244557
	9	progestin.mp. or exp Progestins/	75022
	10	estrogen-progestin.mp.	1246
	11	exp Body Composition/ or body compos*.mp.	613379
	12	1 or 2 or 3 or 4 or 5 or 6 or 7 or 8 or 9 or 10	313714
	13	exp Muscle, Skeletal/ or muscle mass.mp.	263546
	14	11 or 13	318151
	15	12 and 14	4026
**Embase**	1	exp Hormone Replacement Therapy/ or hormon* replacement therap*.mp.	58601
	2	(hormon* adj2 therap*).mp.	83063
	3	(hormon* adj2 replac*).mp.	23065
	4	HRT.mp.	12942
	5	(hormon* adj2 supplement*).mp.	1503
	6	exp Estrogen Replacement Therapy/ or estrogen replacement therap*.mp.	24309
	7	(estrogen adj2 therap*).mp.	26872
	8	estrogen.mp. or exp Estrogens/	338741
	9	progestin.mp. or exp Progestins/	166652
	10	estrogen-progestin.mp.	1325
	11	exp Body Composition/ or body compos*.mp.	88524
	12	1 or 2 or 3 or 4 or 5 or 6 or 7 or 8 or 9 or 10	476514
	13	exp Muscle, Skeletal/ or muscle mass.mp.	295471
	14	11 or 13	373975
	15	12 and 14	7475
**AgeLine**	1	(MH “Hormone Replacement Therapy+”) OR “hormon* replacement therap*”	454
	2	“hrt”	158
	3	“hormon* n2 supplement*”	50
	4	“estrogen replacement therap*”	72
	5	“hormon* n2 therap*”	0
	6	“hormon* n2 replac*”	0
	7	(MH “Estrogens+”) OR “estrogen”	452

©Javed AA et al. *JAMA Network Open*.

	8	“estrogen n2 therap*”	26
	9	(MH “Progestational Hormones+”) OR “progestin”	32
	10	“estrogen-progestin”	14
	11	(MH “Body Composition+”) OR “body compos*”	422
	12	“muscle mass” OR (MH “Muscle, Skeletal+”)	234
	13	S1 or S2 or S3 or S4 or S5 or S6 or S7 or S8 or S9 or S10	749
	14	S11 or S12	605
	15	S13 and S14	16
**CINAHL**	1	(MH “Hormone Replacement Therapy+”) OR “hormon* replacement therap*”	7332
	2	“hrt”	1363
	3	“hormon* n2 supplement*”	0
	4	“estrogen replacement therap*”	315
	5	“hormon* n2 therap*”	0
	6	“hormon* n2 replac*”	0
	7	(MH “Estrogens+”) OR “estrogen”	11378
	8	“estrogen n2 therap*”	0
	9	(MH “Progestational Hormones+”) OR “progestin”	3178
	10	“estrogen-progestin”	211
	11	(MH “Body Composition+”) OR “body compos*”	12555
	12	“muscle mass” OR (MH “Muscle, Skeletal+”)	24568
	13	S1 or S2 or S3 or S4 or S5 or S6 or S7 or S8 or S9 or S10	16947
	14	S11 or S12	35787
	15	S13 and S14	400
**SportDiscus**	1	(MH “Hormone Replacement Therapy+”) OR “hormon* replacement therap*”	388
	2	“hrt”	261
	3	“hormon* n2 supplement*”	1
	4	“estrogen replacement therap*”	60
	5	“hormon* n2 therap*”	0
	6	“hormon* n2 replac*”	1
	7	(MH “Estrogens+”) OR “estrogen”	1856
	8	“estrogen n2 therap*”	0
	9	(MH “Progestational Hormones+”) OR “progestin”	83
	10	“estrogen-progestin”	19
	11	(MH “Body Composition+”) OR “body compos*”	11308
	12	“muscle mass” OR (MH “Muscle, Skeletal+”)	2740
	13	S1 or S2 or S3 or S4 or S5 or S6 or S7 or S8 or S9 or S10	2441
	14	S11 or S12	13527
	15	S13 and S14	102

©Javed AA et al. *JAMA Network Open*.

eTable 2. Estrogen Dose Equivalence Calculations

Reference values[1–3]:

	Estrogen dose for bone endpoints			
**Estrogen**	**Ultra-Low**	**Low**	**Standard**	**High**
Conjugated equine estrogens (mg)	0.15	0.3	0.625	1.25
Micronized 17β-estradiol (mg)	0.5		1[4]	4
Estradiol valerate (mg)		1	2	
Transdermal 17β-estradiol (Estraderm) (mg)[5,6]			0.05	

Calculations:

Study	Estrogen type	Name	Dose	Standardized to CEE's
**Type 1: Conjugated equine estrogens (CEE) (mg)**				
Aloia et al.	E-P[a]	Conjugated equine estrogens and Medroxyprogesterone	E[b]: 0.625 mg<br>P[c]: 10 mg	E: 0.625 mg
Bea et al.	1) E	1) Conjugated equine estrogens (Premarin)	E: 0.625 mg/d	E: 0.625 mg/d
	2) E-P	2) Conjugated equine estrogens and Medroxyprogesterone	E: 0.625 mg/d<br>P: 2.5 mg/d	E: 0.625 mg/d
Chen et al.	E-P	Conjugated equine estrogens and Medroxyprogesterone	E: 0.625 mg/d<br>P: 2.5 mg/d	E: 0.625 mg/d
Evans et al.	E-P	Conjugated equine estrogens and Medroxyprogesterone	E: 0.625 mg/d<br>P: 5 mg/d	E: 0.625 mg/d
Thorneycroft et al.	1) E	1) Conjugated estrogens	a) E: 0.625 mg/d<br>b) E: 0.45 mg/d<br>c) E: 0.3 mg/d	a) E: 0.625 mg/d<br>b) E: 0.45 mg/d<br>c) E: 0.3 mg/d
	2) E-P	2) Conjugated equine estrogens and Medroxyprogesterone	a) E: 0.625 mg/d<br>P: 2.5 mg/d<br>b) E: 0.45 mg/d<br>P: 2.5 mg/d<br>c) E: 0.45 mg/d<br>P: 1.5 mg/d<br>d) E: 0.3 mg/d	a) E: 0.625 mg/d<br>b) E: 0.45 mg/d<br>c) E: 0.45 mg/d<br>d) E: 0.3 mg/d

[a] Estrogen-progesterone
[b] Estrogen
[c] Progesterone

©Javed AA et al. *JAMA Network Open*.

			P: 1.5 mg/d	
**Type 2: Micronized 17 beta estradiol (mg)**				
Hassager & Christiansen	2) E (Percutaneous)	2) 17 beta-estradiol (estrogel cream)	E: 0.6 mg	E: 0.375 mg (1 mg 17b = 0.625 mg CEE à 0.6 mg 17b = 0.375 mg CEE)
Jensen et al.	1) E	1) Oral continuous estradiol (Estrofem)	E: 2 mg/d	E: 1.25 mg/d (1 mg 17b = 0.625 mg CEE à 2 mg 17b = 1.25 mg CEE)
	2) E-P	2) Sequential oral estrogen and progestogen (Trisequens)	E: 2 mg/d<br>P: 1 mg/d	E: 1.25 mg/d (1 mg 17b = 0.625 mg CEE à 2 mg 17b = 1.25 mg CEE)
Kenny et al.	E	17-beta estradiol	E: 0.25 mg/d	E: 0.16 mg (1 mg 17b = 0.625 mg CEE à 0.25 mg 17b = 0.16 mg CEE)
Pöllänen et al.	E-P	Combined estradiol + noretisterone acetate (synthetic progesterone)	E: 2 mg/d<br>P: 1 mg/d	E: 1.25 mg/d (1 mg 17b = 0.625 mg CEE à 2 mg 17b = 1.25 mg CEE)
Sipilä et al.	E-P	Oestradiol and noretisterone acetate (synthetic progesterone) (Kliogest)*this is 17 beta estradiol	E: 2 mg/d<br>P: 1 mg/d	E: 1.25 mg/d (1 mg 17b = 0.625 mg CEE à 2 mg 17b = 1.25 mg CEE)
Sørensen et al.	E-P	17 beta-estradiol and cyclic norethisterone acetate (Trisequens Forte)	E: 4 mg/d<br>P: 1 mg/d	E: 2.5 mg/d (1 mg 17b = 0.625 mg CEE à 4 mg 17b = 2.5 mg CEE)
**Type 3: Estradiol valerate (mg)**				
Haarbo et al.	1) E-P	1) Estradiol valerate + cyproterone acetate (CPA)	E: 2 mg/d<br>P: 1 mg/d	E: 0.625 mg/d
	2) E-P	2) Estradiol valerate + levonorgestrel (LNG)	E: 2 mg/d	E: 0.625 mg/d

			P: 75 µg/d	
Hassager & Christiansen	1) E-P (Oral)	1) Estradiol valerate + cyproterone acetate	E: 2 mg/d<br>P: 1 mg	E: 0.625 mg/d
**Type 4: Transdermal estradiol**				
Blackman et al.	E-P	Estradiol transdermal patches (Estraderm) +medroxyprogesterone acetate (Provera)	E: 100 µg/d<br>à(0.1 mg)<br>P: 10 mg/d	E: 1.25 mg/d<br>(0.05 mg = 0.625 mg CEE à0.1 mg = 1.25 mg CEE)

References

1. Gambacciani M, Genazzani AR. Hormone replacement therapy: The benefits in tailoring the regimen and dose. *Maturitas*. 2001;40(3):195-201. doi:10.1016/S0378-5122(01)00281-X
2. Lindsay R, Hart DM, Clark DM. The minimum effective dose of estrogen for prevention of postmenopausal bone loss. *Obstet Gynecol*. 1984;63(6):759-763.
3. Panay N, Ylikorkala O, Archer DF, Gut R, Lang E. Ultra-low-dose estradiol and norethisterone acetate: Effective menopausal symptom relief. *Climacteric*. 2007;10(2):120-131. doi:10.1080/13697130701298107
4. Archer DF. Estrace® vs Premarin® for treatment of menopausal symptoms: Dosage comparison study. *Adv Ther*. 1992;9:21-31.
5. Anderson PO, Knoben JE, Troutman WG. *Handbook of Clinical Drug Data*.; 2002. doi:10.7326/0003-4819-110-11-948_2
6. Kenigsberg L, Balachandar S, Prasad K, Shah B. Exogenous Pubertal Induction by Oral versus Transdermal Estrogen Therapy. *J Pediatr Adolesc Gynecol*. 2013;26(2):71-79. doi:10.1016/j.jpag.2011.09.012

©Javed AA et al. *JAMA Network Open*.

eTable 3. Study Characteristics (Part 1)

Reference	Publication Date	Study Date	Country	Total recruited participants	Total participants included in analysis	Age (years)	Ethnicity
Aloia et al.	1995	N/A	USA	118	77	52.162 ± 0.654	Caucasian
Bea et al.	2001	1993-2004	USA	1) 927<br>2) 1014	1) 927<br>2) 1014	1) 63.35 ± 7.6<br>2) 63.29 ± 7.2	N/A
Blackman et al.	2002	1992-1998	USA	28	28	71.5 ± 1.12	N/A
Chen et al.	2005	1993-2001	USA	835	835<br>***Sensitivity analysis:*** 511 (256 placebo, 255 treatment)	63.1 ± 7.2	Caucasian 82.4%<br>Black 10.4%<br>Hispanic 5.3%<br>American Indian 1.07%<br>Asian or Pacific Islander 0.35%<br>Other or unknown 0.48%
Evans et al.	2001	N/A	USA	68	68 (But only 34 in HT and placebo groups)	67.7 ± 5.2	N/A
Haarbo et al.	1991	N/A	Denmark	75	1) 43 (19 treatment, 24 placebo)<br>2) 43 (19 treatment, 24 placebo)	45-55 years	N/A
Hassager & Christiansen	1989	1983-1985	Denmark	133	1) 65 (32 treatment, 33 control)<br>2) 45 (20 treatment, 25 control)	1) 49.91 ± 2.36<br>2) 50.41 ± 2.29	N/A
Jensen et al.	2003	1990-1993	Denmark	1006 (502 treatment, 504 placebo)	621 (268 treatment, 353 placebo)	50.1 ± 2.8	N/A
Kenny et al.	2005	N/A	USA	167	107 (At follow up 58 - treatment, 49 - placebo)	74.3 ± 0.6	N/A
Pöllänen et al.	2007	N/A	Finland	20	15	53.6 ± 1.85	N/A
Sipilä et al.	2001	N/A	Finland	80	52 (But only 30 in HT and placebo groups)	50-55	N/A
Sørensen et al.	2001	N/A	Denmark	16	14	55.5 ± 2.6	N/A
Thorneycroft et al.	2007	N/A	USA	822	502	51.6 ± 3.7	Caucasian 90%<br>Other 10%

eTable 4. Study Characteristics (Part 2)

**Reference** *(continued)*	**Type of Menopause**	**Mean time since menopause + SE**	**HT**	[illegible]	[illegible]
Aloia et al.	Natural	2.27±0.33 years	E-P	E: 0.625 mg<br>P: 10 mg	E: 25 days out of a month<br>P: 9 days (days 16 - 25)
Bea et al.	Both (Baseline - 47.7% were induced, 52.2% natural)	1) 22.21 ± 8.4 years	1) E	0.625 mg/d	7.7±1.8 years
		2) 13.53 ± 8.5 years	2) E-P	E: 0.625 mg/d<br>P: 2.5 mg/d	6.3±1.5 years
Blackman et al.	N/A	N/A	E-P	E: 100 µg/d for 6 months<br>P: 10 mg/d for	E: 6 months<br>P: Last 10 days of each 28-day cycle for 6 months (~60-65 days)
Chen et al.	Natural	13.8 ± 8.9 years	E-P	E: 0.625 mg/d<br>P: 2.5 mg/d	3 years
Evans et al.	N/A	Mean age at menopause: 49 ± 5, Current mean age: 67.8 ± 5	E-P	E: 0.625 mg/d<br>P: 5 mg/d	13 days every 3rd month
Haarbo et al.	Natural	20.08 ± 8.97 months	1) E-P	E: 2 mg/d<br>P: 1 mg/d	2 years
			2) E-P	E: 2 mg/d<br>P: 75 µg/d	2 years
Hassager & Christiansen	Natural	Inclusion criteria: menopause within the last 0.5-3 years	1) E-P (Oral)	E: 2 mg/d<br>P: 1 mg	In a 28-day cycle: E: days 1-11<br>E-P: days 12-21<br>None: days 22-28
			2) E (Percutaneous)	E: 0.6 mg	In a 28-day cycle,<br>E: days 1-24, 5 g<br>None: days 25-28
Jensen et al.	Both (Baseline - 41% were induced, 59% natural)	0.7 ± 0.6 years	1) E	2 mg/d	5 years
			2) E-P	E: 2 mg<br>P: 1 mg	In a 28 day cycle:<br>E: days 1-12<br>E-P: days 13-22 E: days 23-28
Kenny et al.	N/A	Mean age: 74.3 ± 0.6 (older postmenopausal)	E	0.25 mg/d (ultra-low dose)	36 months
Pöllänen et al.	N/A	2.8 ± 3.6 years	E-P	E: 2 mg/d<br>P: 1 mg/d	1 year
Sipilä et al.	N/A	Inclusion criteria: menopause within the last 5 years	E-P	E: 2 mg/d<br>P: 1 mg/d	1 year
Sørensen et al.	Natural	5.9 ± 3.9 years	E-P	E: 4 mg<br>P: 1 mg	In a 28 day cycle:<br>E: 4 mg for 22 days and 1 mg for 6 days, P: 10 days. Total 12 weeks
Thorneycroft et al.	Natural	2.3 ± 0.9 years	1) E	a) E: 0.625 mg/d	2 years
				b) E: 0.45 mg/d	2 years
				c) E: 0.3 mg/d	2 years
			2) E-P	a) E: 0.625 mg/d<br>P: 2.5 mg/d	2 years
				b) E: 0.45 mg/d<br>P: 2.5 mg/d	2 years
				c) E: 0.45 mg/d<br>P: 1.5 mg/d	2 years
				d) E: 0.3 mg/d<br>P: 1.5 mg/d	2 years

©Javed AA et al. *JAMA Network Open*.

eTable 5. Study Characteristics (Part 3)

**Reference** *(continued)*	**HT** *(continued)*		**Comparison Groups**	**Follow-up period**
	**Name**	**Cyclical or Continuous?**	**Placebo or Control?**	
Aloia et al.	Conjugated equine estrogens and Medroxyprogesterone	Continuous	Placebo	2.9 ± 1.1 years
Bea et al.	1) Conjugated equine estrogens (Premarin)	Continuous	Placebo	6 years
	2) Conjugated equine estrogens and Medroxyprogesterone	Continuous		
Blackman et al.	Estradiol transdermal patches (Estraderm) + medroxyprogesterone acetate (Provera)	Continuous	Placebo	6 months
Chen et al.	Conjugated equine estrogens and Medroxyprogesterone	Continuous	Placebo	3 years
Evans et al.	Conjugated equine estrogens and Medroxyprogesterone	Cyclical	Control	N/A
Haarbo et al.	1) Estradiol valerate + cyproterone acetate (CPA)	Continuous	Placebo	2 years
	2) Estradiol valerate + levonorgestrel (LNG)	Continuous		
Hassager & Christiansen	1) Estradiol valerate + cyproterone acetate	Cyclical	Control	2 years
	2) 17 beta-estradiol (estrogel cream)	Continuous		
Jensen et al.	1) Oral continuous estradiol (Estrofem)	Continuous	Control	5 years
	2) Sequential oral estrogen and progestogen (Trisequens)	Cyclical		
Kenny et al.	17-beta estradiol	Continuous	Placebo	3 years
Pöllänen et al.	Combined estradiol + noretisterone acetate (synthetic progesterone)	Continuous	Placebo	12 months
Sipilä et al.	Oestradiol and noretisterone acetate (synthetic progesterone) (Kliogest)	Continuous	Placebo	12 months
Sørensen et al.	17 beta-estradiol and cyclic norethisterone acetate (Trisequens Forte)	Cyclical	Placebo	N/A
Thorneycroft et al.	1) Conjugated estrogens	a) Continuous	Placebo	2 years
		b) Continuous		
		c) Continuous		
	2) Conjugated equine estrogens and Medroxyprogesterone	a) Continuous		
		b) Continuous		
		c) Continuous		
		d) Continuous		

©Javed AA et al. *JAMA Network Open*.

eTable 6. Risk of Bias Assessment

	**Selection bias:** Random sequence generation	**Selection bias:** Allocation concealment	**Performance bias:** Blinding of participants and personnel	**Detection bias:** Blinding of outcome assessment	**Attrition bias:** Incompl-ete outcome data	**Reporting bias:** Selective Reporting	**Other bias:** Other sources of bias	**Overall Risk of Bias?**
Aloia et al.	Low	Unclear[1]	Unclear[1]	Low[2]	High[3]	High[4]	High[5]	**High**
Bea et al.	Low	Unclear	Low	Low	Low	Low	Low	**Unclear**
Blackman et al.	Low	Unclear[1]	Low	Low	Low	Low	High	**Unclear**[6]
Evans et al.	High	Unclear	Unclear	Unclear	Low	Low	Low	**High**
Haarbo et al.	Low	Unclear	Unclear	Unclear	High	Low	High[7]	**High**
Hassager & Christiansen	Low	Unclear	Low[8]	Low	Low[9]	High[10]	Low	**Unclear**[6]
Jensen et al.	Low[11]	Low	High	High	Low[12]	Low	Low	**High**
Kenny et al.	Low	Unclear	Low	Low	Low	High[13]	High[14]	**High**
Pöllänen et al.	Low	Unclear	Low	Low	High[15]	Low	High[16]	**High**[7]
Sipilä et al.	Low	Low	Low	Low	Low	Low	Low	**Low**
Sørensen et al.	Low	Low	Low	Low	Low	Low	High[18]	**Unclear**
Thorneycroft et al.	Low	Unclear	Low	Low	Low[19]	Low	Low	**Low**

[1] No information. This was never stated implicitly or explicitly.

[2] If it is double blind, we can assume that this is low risk.

[3] Study did not explain which groups the women dropped out of, although they gave reasons.

[4] The 'per year' analysis warranted a "high" assessment.

[5] Control group received vitamin D, and there are potentially unbalanced baseline groups.

[6] The "unclear" category is unlikely to bias the outcome.

[7] Menopausal age was very different in the placebo group.

[8] One treatment arm affected by unblinding, but it is reasonable.

[9] Percutaneous group has higher rates of dropout due to side effects, but they did report the outcomes.

[10] Combined placebo groups and didn't explain why, nor quantify the similarity.

[11] Only partial randomization, but we only considered the randomized groups.

[12] Didn't give reasons for dropout, but the numbers are balanced. Used intention to treat analysis.

[13] No results of mixed model reported, although they mentioned it.

[14] Progesterone was given to placebo women as well.

[15] Did not address reasons for dropout, which is quite different between groups.

[16] Very small study.

[17] This study was not designed for our purposes.

[18] Very short follow-up.

[19] Small percentage of dropout.

eTable 7. Summary Meta-analysis of the Association Between Less Than 0.625 mg Estrogen-Only Treatment and Muscle Mass Outcomes

Impact of Low Dose Estrogen on Lean Body Mass by Study

Study Name	Experimental		Control		Mean Difference	95% CI
	**Mean + SD**	**Total**	**Mean + SD**	**Total**		
Hassager, 1989	2) 0.81±1.65	20	0.33±2.13	58	0.48	-0.43, 1.39
Kenny, 2005	-0.3±3.37	71	-0.5±3.3	68	0.20	-0.91, 1.31
Thorneycroft, 2007	1b) 0.26±1.56	95	0.19±1.55	94	0.07	-0.37, 0.51
	1c) -0.04±1.51	89	0.19±1.55	94	-0.23	-0.67, 0.21
**Total**		**275**		**314**	**-0.01**	**-0.29, 0.28**
Heterogeneity	Chi^2= 2.33, df =3 (P=0.51); I^2=0%					
Test for overall effect	Z=0.04 (P=0.97)					

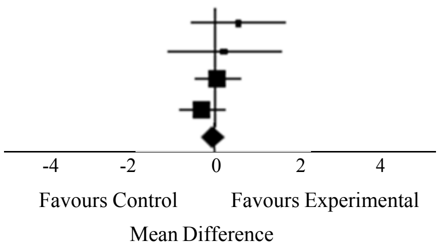

Caption: The forest plot of the meta-analyses of treatment arms utilizing less than 0.625 mg estrogen-only treatment, which presents the mean differences and 95% confidence intervals for lean body mass between women taking hormone replacement therapy and women who were not taking hormone replacement therapy (control group).

eTable 8. Summary Meta-analysis of the Association Between 0.625 mg or More Estrogen-Only Treatment and Muscle Mass Outcomes

Impact of High Dose Estrogen on Lean Body Mass by Study

Study Name	Experimental		Control		Mean Difference	95% CI
	**Mean + SD**	**Total**	**Mean + SD**	**Total**		
Thorneycroft, 2007	1a) -0.12±1.87	97	0.19±1.55	94	-0.31	-0.80, 0.18
Bea, 2001	1) -0.44±2.28	453	-0.5±2.45	474	0.06	-0.24, 0.36
**Total**		**550**		**568**	**-0.04**	**-0.30, 0.21**
Heterogeneity	Chi^2= 1.60, df =1 (P=0.21); I^2=37%					
Test for overall effect	Z=0.34 (P=0.74)					

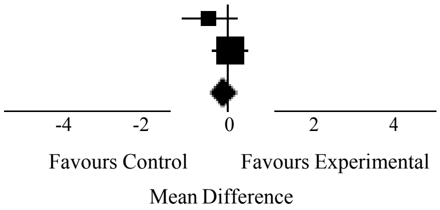

Caption: The forest plot of the meta-analyses of treatment arms utilizing 0.625 mg or more estrogen-only treatment, which presents the mean differences and 95% confidence intervals for lean body mass between women taking hormone replacement therapy and women who were not taking hormone replacement therapy (control group).

©Javed AA et al. *JAMA Network Open*.

eTable 9. Summary Meta-analysis of the Association Between Less than 0.625 mg Estrogen + Any Dose Progesterone Treatment and Muscle Mass Outcomes

Impact of Low Dose Estrogen-Progesterone on Lean Body Mass by Study

Study Name	Experimental		Control		Mean Difference	95% CI
	**Mean + SD**	**Total**	**Mean + SD**	**Total**		
Thorneycroft, 2007	2b) 0.1±1.47	96	0.19±1.55	94	-0.09	-0.52, 0.34
	2c) 0.13±1.45	94	0.19±1.55	94	-0.06	-0.49, 0.37
	2d) 0.16±1.39	98	0.19±1.55	94	-0.03	-0.45, 0.39
**Total**		**288**		**282**	**-0.06**	**-0.30, 0.19**
Heterogeneity	Chi^2= 0.04, df =2 (P=0.98); I^2=0%					
Test for overall effect	Z=0.47 (P=0.64)					

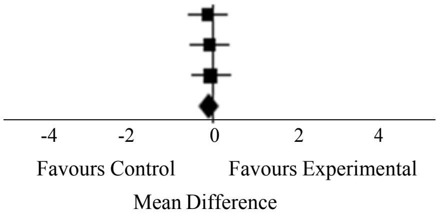

Caption: The forest plot of the meta-analyses of treatment arms utilizing less than 0.625 mg estrogen + any dose progesterone treatment, which presents the mean differences and 95% confidence intervals for lean body mass between women taking hormone replacement therapy and women who were not taking hormone replacement therapy (control group).

©Javed AA et al. *JAMA Network Open*.

eTable 10. Summary Meta-analysis of the Association Between 0.625 mg or More Estrogen + Any Dose Progesterone Treatment and Muscle Mass Outcomes

Impact of High Dose Estrogen-Progesterone on Lean Body Mass by Study

**Study Name**	**Experimental**		**Control**		**Mean Difference**	**95% CI**
	**Mean + SD**	**Total**	**Mean + SD**	**Total**		
Hassager, 1989	1) 0.19±2.15	32	0.33±2.13	58	-0.14	-1.06, 0.78
Haarbo, 1991	1) -0.2±5.2	19	-0.7±3.85	24	0.5	-2.30, 3.30
	2) 0.6±3.7	19	-0.7±3.85	24	1.30	-1.00, 3.60
Aloia, 1995	-1.06±1.64	30	-0.75±1.59	28	-0.31	-1.14, 0.52
Evans, 2001	1.1±1.9	15	0.5±1.4	19	0.60	-0.55, 1.75
Sipilä, 2001	1.1±4.25	15	-0.3±4.65	15	1.40	-1.79, 4.59
Sørensen, 2001	0.35±0.86	7	-1.0±1.58	7	1.34	0.01, 2.67
Blackman, 2002	1.2±4.58	19	0.4±3.93	14	0.8	-2.11, 3.71
Pöllänen, 2007	1.00±4.00	10	-1.4±3.10	5	2.40	-1.28, 6.08
Thorneycroft, 2007	2a) 0.55±1.48	86	0.19±1.55	94	0.36	-0.08, 0.80
Bea, 2001	2) -0.29±1.99	543	-0.4±2.15	471	0.11	-0.15, 0.37
**Total**		**795**		**759**	**0.19**	**-0.01, 0.39**
Heterogeneity	Chi^2= 9.23, df =10 (P=0.51); I^2=0%					
Test for overall effect	Z=1.90 (P=0.06)					

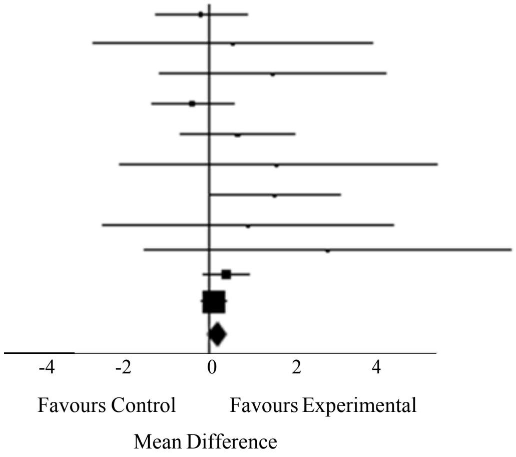

Caption: The forest plot of the meta-analyses of treatment arms utilizing 0.625 mg or more estrogen + any dose progesterone treatment, which presents the mean differences and 95% confidence intervals for lean body mass between women taking hormone replacement therapy and women who were not taking hormone replacement therapy (control group).

©Javed AA et al. *JAMA Network Open*.

eTable 11. Summary Meta-analysis of the Association Between Shorter Follow-up Lengths and Muscle Mass Outcomes

Impact of Shorter Follow-Ups on Lean Body Mass by Study

Study Name	Experimental		Control		Mean Difference	95% CI
	**Mean + SD**	**Total**	**Mean + SD**	**Total**		
Hassager, 1989	1) 0.19±2.15	32	0.33±2.13	58	-0.14	-1.06, 0.78
	2) 0.81±1.65	20	0.33±2.13	58	0.48	-0.43, 1.39
Haarbo, 1991	1) -0.2±5.2	19	-0.7±3.85	24	0.5	-2.30, 3.30
	2) 0.6±3.7	19	-0.7±3.85	24	1.30	-1.00, 3.60
Sipilä, 2001	1.1±4.25	15	-0.3±4.65	15	1.40	-1.79, 4.59
Blackman, 2002	1.2±4.58	19	0.4±3.93	14	0.8	-2.11, 3.71
Pöllänen, 2007	1.00±4.00	10	-1.4±3.10	5	2.40	-1.28, 6.08
Thorneycroft, 2007	1a) -0.12±1.87	97	0.19±1.55	94	-0.31	-0.80, 0.18
	1b) 0.26±1.56	95	0.19±1.55	94	0.07	-0.37, 0.51
	1c) -0.04±1.51	89	0.19±1.55	94	-0.23	-0.67, 0.21
	2a) 0.55±1.48	86	0.19±1.55	94	0.36	-0.08, 0.80
	2b) 0.1±1.47	96	0.19±1.55	94	-0.09	-0.52, 0.34
	2c) 0.13±1.45	94	0.19±1.55	94	-0.06	-0.49, 0.37
	2d) 0.16±1.39	98	0.19±1.55	94	-0.03	-0.45, 0.39
**Total**		**789**		**856**	**0.00**	**-0.16, 0.16**
Heterogeneity	Chi^2= 10.67, df =13 (P=0.64); I^2=0%					
Test for overall effect	Z=0.04 (P=0.97)					

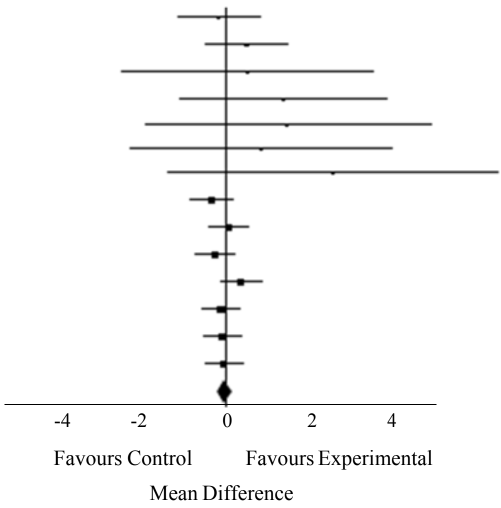

Caption: The forest plot of the meta-analyses of studies with shorter follow-up lengths, which presents the mean differences and 95% confidence intervals for lean body mass between women taking hormone replacement therapy and women who were not taking hormone replacement therapy (control group).

eTable 12. Summary Meta-analysis of the Association Between Longer Follow-up Lengths and Muscle Mass Outcomes

Impact of Longer Follow-Ups on Lean Body Mass by Study

Study Name	Experimental		Control		Mean Difference	95% CI
	**Mean + SD**	**Total**	**Mean + SD**	**Total**		
Aloia, 1995	-1.06±1.64	30	-0.75±1.59	28	-0.31	-1.14, 0.52
Jensen, 2003	0.18±1.77	268	-0.02±2.33	353	0.20	-0.12, 0.52
Kenny, 2005	-0.3±3.37	71	-0.5±3.3	68	0.20	-0.91, 1.31
Bea, 2001	1) -0.44±2.28	453	-0.5±2.45	474	0.06	-0.24, 0.36
	2) -0.29±1.99	543	-0.4±2.15	471	0.11	-0.15, 0.37
**Total**		**1365**		**1394**	**0.10**	**-0.06, 0.27**
Heterogeneity	Chi^2= 1.40, df =4 (P=0.84); I^2=0%					
Test for overall effect	Z=1.26 (P=0.21)					

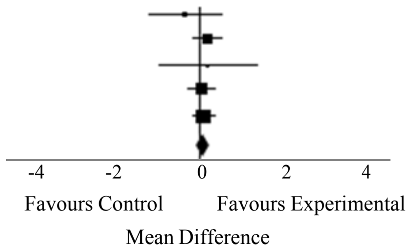

Caption: The forest plot of the meta-analyses of studies with longer follow-up lengths, which presents the mean differences and 95% confidence intervals for lean body mass between women taking hormone replacement therapy and women who were not taking hormone replacement therapy (control group).

©Javed AA et al. *JAMA Network Open*.

eTable 13. Summary Meta-analysis of Studies With <10 Years of Time Since Menopause

Impact of Younger Menopausal Age on Lean Body Mass by Study

Study Name	Experimental		Control		Mean Difference	95% CI
	Mean + SD	Total	Mean + SD	Total		
Hassager, 1989	1) 0.19±2.15	32	0.33±2.13	58	-0.14	-1.06, 0.78
	2) 0.81±1.65	20	0.33±2.13	58	0.48	-0.43, 1.39
Haarbo, 1991	1) -0.2±5.2	19	-0.7±3.85	24	0.5	-2.30, 3.30
	2) 0.6±3.7	19	-0.7±3.85	24	1.30	-1.00, 3.60
Aloia, 1995	-1.06±1.64	30	-0.75±1.59	28	-0.31	-1.14, 0.52
Sipilä, 2001	1.1±4.25	15	-0.3±4.65	15	1.40	-1.79, 4.59
Sørensen, 2001	0.35±0.86	7	-1.0±1.58	7	1.34	0.01, 2.67
Jensen, 2003	0.18±1.77	268	-0.02±2.33	353	0.20	-0.12, 0.52
Pöllänen, 2007	1.00±4.00	10	-1.4±3.10	5	2.40	-1.28, 6.08
Thorneycroft, 2007	1a) -0.12±1.87	97	0.19±1.55	94	-0.31	-0.80, 0.18
	1b) 0.26±1.56	95	0.19±1.55	94	0.07	-0.37, 0.51
	1c) -0.04±1.51	89	0.19±1.55	94	-0.23	-0.67, 0.21
	2a) 0.55±1.48	86	0.19±1.55	94	0.36	-0.08, 0.80
	2b) 0.1±1.47	96	0.19±1.55	94	-0.09	-0.52, 0.34
	2c) 0.13±1.45	94	0.19±1.55	94	-0.06	-0.49, 0.37
	2d) 0.16±1.39	98	0.19±1.55	94	-0.03	-0.45, 0.39
**Total**		**1075**		**1230**	**0.04**	**-0.10, 0.18**
Heterogeneity	Chi^2= 15.99, df = 15 (P=0.38); I^2=6%					
Test for overall effect	Z= 0.55 (P=0.58)					

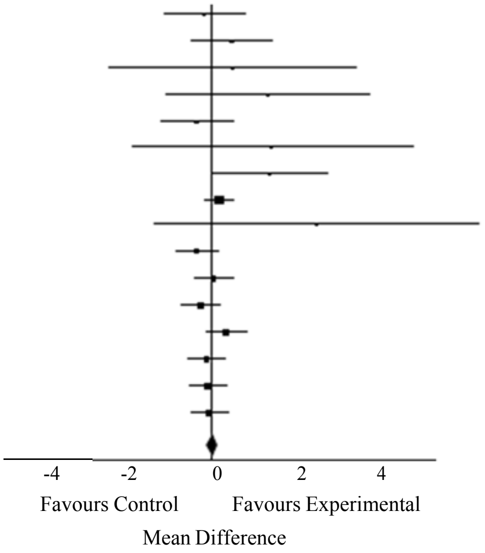

Caption: The forest plot of the meta-analyses of studies with participants who had <10 years of time since menopause, presenting the mean differences and 95% confidence intervals for lean body mass between women taking hormone replacement therapy and women who were not taking hormone replacement therapy (control group).

©Javed AA et al. *JAMA Network Open*.

eTable 14. Summary Meta-analysis of the Association Between Shorter Times Since Menopause and Muscle Mass Outcomes

Impact of Shorter Time Since Menopause on Lean Body Mass by Study

**Study Name**	**Experimental**		**Control**		**Mean Difference**	**95% CI**
	**Mean + SD**	**Total**	**Mean + SD**	**Total**		
Haarbo, 1991	1) -0.2±5.2	19	-0.7±3.85	24	0.5	-2.30, 3.30
	2) 0.6±3.7	19	-0.7±3.85	24	1.30	-1.00, 3.60
Aloia, 1995	-1.06±1.64	30	-0.75±1.59	28	-0.31	-1.14, 0.52
Jensen, 2003	0.18±1.77	268	-0.02±2.33	353	0.20	-0.12, 0.52
Pöllänen, 2007	1.00±4.00	10	-1.4±3.10	5	2.40	-1.28, 6.08
Thorneycroft, 2007	1a) -0.12±1.87	97	0.19±1.55	94	-0.31	-0.80, 0.18
	1b) 0.26±1.56	95	0.19±1.55	94	0.07	-0.37, 0.51
	1c) -0.04±1.51	89	0.19±1.55	94	-0.23	-0.67, 0.21
	2a) 0.55±1.48	86	0.19±1.55	94	0.36	-0.08, 0.80
	2b) 0.1±1.47	96	0.19±1.55	94	-0.09	-0.52, 0.34
	2c) 0.13±1.45	94	0.19±1.55	94	-0.06	-0.49, 0.37
	2d) 0.16±1.39	98	0.19±1.55	94	-0.03	-0.45, 0.39
**Total**		**1001**		**1092**	**0.01**	**-0.13, 0.16**
Heterogeneity	Chi^2= 10.45, df =11 (P=0.49); I^2=0%					
Test for overall effect	Z=0.20 (P=0.84)					

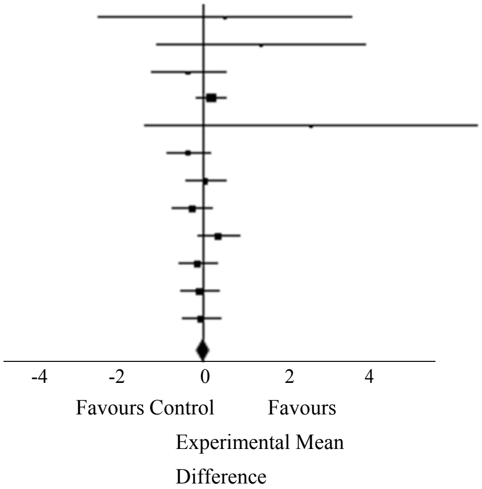

Caption: The forest plot of the meta-analyses of studies with shorter times since menopause, which presents the mean differences and 95% confidence intervals for lean body mass between women taking hormone replacement therapy and women who were not taking hormone replacement therapy (control group).

©Javed AA et al. *JAMA Network Open*.

eTable 15. Summary Meta-analysis of the Association Between Longer Times Since Menopause and Muscle Mass Outcomes

Impact of Longer Time Since Menopause on Lean Body Mass by Study

Study Name	Experimental		Control		Mean Difference	95% CI
	**Mean + SD**	**Total**	**Mean + SD**	**Total**		
Evans, 2001	1.1±1.9	15	0.5±1.4	19	0.60	-0.55, 1.75
Sørensen, 2001	0.35±0.86	7	-1.0±1.58	7	1.34	0.01, 2.67
Bea, 2001	1) -0.44±2.28	453	-0.5±2.45	474	0.06	-0.24, 0.36
	2) -0.29±1.99	543	-0.4±2.15	471	0.11	-0.15, 0.37
**Total**		**1018**		**971**	**0.13**	**-0.06, 0.32**
Heterogeneity	Chi^2= 4.05, df =3 (P=0.26); I^2=26%					
Test for overall effect	Z=1.32 (P=0.19)					

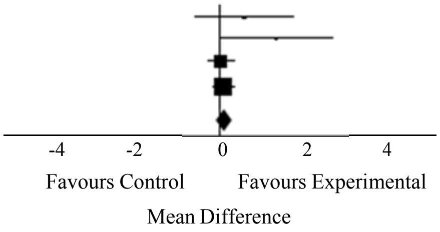

Caption: The forest plot of the meta-analyses of studies with longer times since menopause, which presents the mean differences and 95% confidence intervals for lean body mass between women taking hormone replacement therapy and women who were not taking hormone replacement therapy (control group).

©Javed AA et al. *JAMA Network Open*.

eTable 16. Summary Meta-analysis of the Association Between Fair/Good Study Quality and Muscle Mass Outcomes

Impact of Better Study Quality on Lean Body Mass by Study

Study Name	Experimental		Control		Mean Difference	95% CI
	Mean + SD	Total	Mean + SD	Total		
Hassager, 1989	1) 0.19±2.15	32	0.33±2.13	58	-0.14	-1.06, 0.78
	2) 0.81±1.65	20	0.33±2.13	58	0.48	-0.43, 1.39
Sipilä, 2001	1.1±4.25	15	-0.3±4.65	15	1.40	-1.79, 4.59
Sørensen, 2001	0.35±0.86	7	-1.0±1.58	7	1.34	0.01, 2.67
Blackman, 2002	1.2±4.58	19	0.4±3.93	14	0.8	-2.11, 3.71
Thorneycroft, 2007	1a) -0.12±1.87	97	0.19±1.55	94	-0.31	-0.80, 0.18
	1b) 0.26±1.56	95	0.19±1.55	94	0.07	-0.37, 0.51
	1c) -0.04±1.51	89	0.19±1.55	94	-0.23	-0.67, 0.21
	2a) 0.55±1.48	86	0.19±1.55	94	0.36	-0.08, 0.80
	2b) 0.1±1.47	96	0.19±1.55	94	-0.09	-0.52, 0.34
	2c) 0.13±1.45	94	0.19±1.55	94	-0.06	-0.49, 0.37
	2d) 0.16±1.39	98	0.19±1.55	94	-0.03	-0.45, 0.39
Bea, 2001	1) -0.44±2.28	453	-0.5±2.45	474	0.06	-0.24, 0.36
	2) -0.29±1.99	543	-0.4±2.15	471	0.11	-0.15, 0.37
**Total**		**1744**		**1755**	**0.04**	**-0.09, 0.16**
Heterogeneity	Chi^2= 12.09, df =13 (P=0.52); I^2=0%					
Test for overall effect	Z=0.60 (P=0.55)					

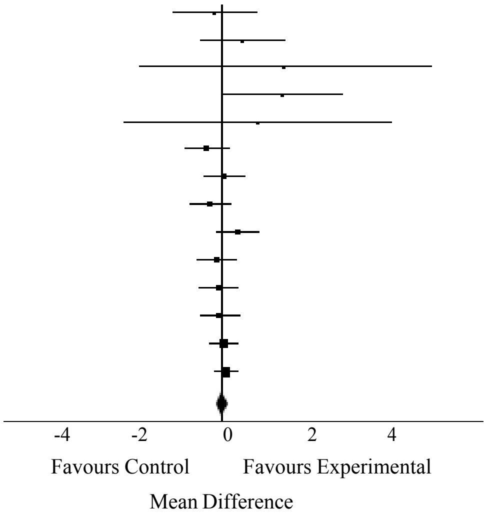

Caption: The forest plot of the meta-analyses of fair/good quality studies, which presents the mean differences and 95% confidence intervals for lean body mass between women taking hormone replacement therapy and women who were not taking hormone replacement therapy (control group).

©Javed AA et al. *JAMA Network Open*.

eTable 17. Summary Meta-analysis of the Association Between Poor Study Quality and Muscle Mass Outcomes

Impact of Poorer Study Quality on Lean Body Mass by Study

**Study Name**	**Experimental**		**Control**		**Mean Difference**	**95% CI**
	**Mean + SD**	**Total**	**Mean + SD**	**Total**		
Haarbo, 1991	1) -0.2±5.2	19	-0.7±3.85	24	0.5	-2.30, 3.30
	2) 0.6±3.7	19	-0.7±3.85	24	1.30	-1.00, 3.60
Aloia, 1995	-1.06±1.64	30	-0.75±1.59	28	-0.31	-1.14, 0.52
Evans, 2001	1.1±1.9	15	0.5±1.4	19	0.60	-0.55, 1.75
Jensen, 2003	0.18±1.77	268	-0.02±2.33	353	0.20	-0.12, 0.52
Kenny, 2005	-0.3±3.37	71	-0.5±3.3	68	0.20	-0.91, 1.31
Pöllänen, 2007	1.00±4.00	10	-1.4±3.10	5	2.40	-1.28, 6.08
**Total**		**432**		**521**	**0.20**	**-0.08, 0.48**
Heterogeneity	Chi^2= 4.21, df =6 (P=0.65); I^2=0%					
Test for overall effect	Z=1.40 (P=0.16)					

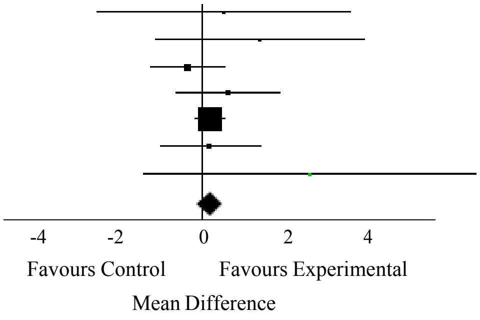

Caption: The forest plot of the meta-analyses of poor quality studies, which presents the mean differences and 95% confidence intervals for lean body mass between women taking hormone replacement therapy and women who were not taking hormone replacement therapy (control group).

©Javed AA et al. *JAMA Network Open*.

eTable 18. Summary Meta-analysis of the Association Between DEXA Measurement and Muscle Mass Outcomes

Impact of Use of DEXA Measurement on Lean Body Mass by Study

Study Name	Experimental		Control		Mean Difference	95% CI
	**Mean + SD**	**Total**	**Mean + SD**	**Total**		
Evans, 2001	1.1±1.9	15	0.5±1.4	19	0.60	-0.55, 1.75
Sørensen, 2001	0.35±0.86	7	-1.0±1.58	7	1.34	0.01, 2.67
Blackman, 2002	1.2±4.58	19	0.4±3.93	14	0.8	-2.11, 3.71
Jensen, 2003	0.18±1.77	268	-0.02±2.33	353	0.20	-0.12, 0.52
Kenny, 2005	-0.3±3.37	71	-0.5±3.3	68	0.20	-0.91, 1.31
Thorneycroft, 2007	1a) -0.12±1.87	97	0.19±1.55	94	-0.31	-0.80, 0.18
	1b) 0.26±1.56	95	0.19±1.55	94	0.07	-0.37, 0.51
	1c) -0.04±1.51	89	0.19±1.55	94	-0.23	-0.67, 0.21
	2a) 0.55±1.48	86	0.19±1.55	94	0.36	-0.08, 0.80
	2b) 0.1±1.47	96	0.19±1.55	94	-0.09	-0.52, 0.34
	2c) 0.13±1.45	94	0.19±1.55	94	-0.06	-0.49, 0.37
	2d) 0.16±1.39	98	0.19±1.55	94	-0.03	-0.45, 0.39
Bea, 2001	1) -0.44±2.28	453	-0.5±2.45	474	0.06	-0.24, 0.36
	2) -0.29±1.99	543	-0.4±2.15	471	0.11	-0.15, 0.37
**Total**		**2031**		**2064**	**0.06**	**-0.06, 0.18**
Heterogeneity	Chi^2= 12.17, df =13 (P=0.51); I^2=0%					
Test for overall effect	Z=1.01 (P=0.31)					

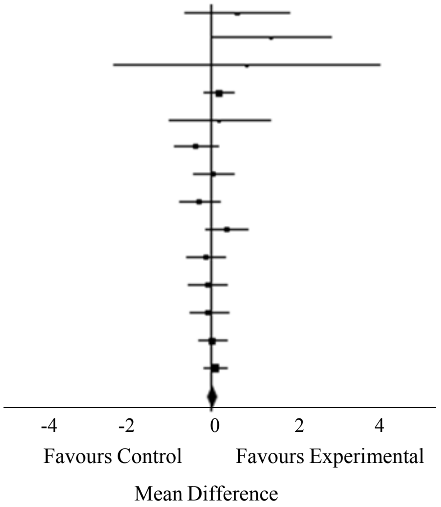

Caption: The forest plot of the meta-analyses of treatment arms utilizing DEXA measurement for muscle mass, which presents the mean differences and 95% confidence intervals for lean body mass between women taking hormone replacement therapy and women who were not taking hormone replacement therapy (control group).

©Javed AA et al. *JAMA Network Open*.

eTable 19. Summary Meta-analysis of the Association Between Other Measurement and Muscle Mass Outcomes

Impact of Use of Other Measurement Types on Lean Body Mass by Study

**Study Name**	**Experimental**		**Control**		**Mean Difference**	**95% CI**
	**Mean + SD**	**Total**	**Mean + SD**	**Total**		
Hassager, 1989	1) 0.19±2.15	32	0.33±2.13	58	-0.14	-1.06, 0.78
	2) 0.81±1.65	20	0.33±2.13	58	0.48	-0.43, 1.39
Aloia, 1995	-1.06±1.64	30	-0.75±1.59	28	-0.31	-1.14, 0.52
Sipilä, 2001	1.1±4.25	15	-0.3±4.65	15	1.40	-1.79, 4.59
Pöllänen, 2007	1.00±4.00	10	-1.4±3.10	5	2.40	-1.28, 6.08
**Total**		**107**		**164**	**0.07**	**-0.43, 0.57**
Heterogeneity	Chi^2= 3.99, df =4 (P=0.41); I^2=0%					
Test for overall effect	Z=0.28 (P=0.78)					

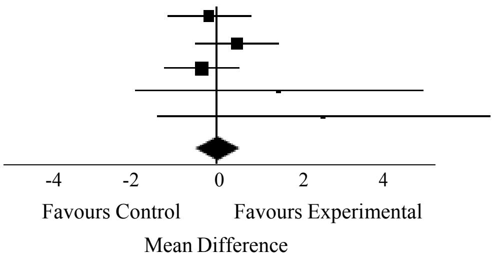

Caption: The forest plot of the meta-analyses of treatment arms utilizing other measurement of muscle mass, which presents the mean differences and 95% confidence intervals for lean body mass between women taking hormone replacement therapy and women who were not taking hormone replacement therapy (control group).

eTable 20. GRADE Assessment

Quality Assessment									Summary of Findings
**Outcome**	**Exposure**	**Participants (# of Studies)**	**Risk of Bias**	**Inconsistency**	**Indirectness**	**Imprecision**[1]	**Publication Bias**	**Quality of Evidence**	**Mean Difference (kg)**
Lean body mass (kg)	E-only or E-P HRT	4452 (12)[2]	Serious[3]	Not serious (I^2=0%)	Not serious	Serious[4]	Serious[5]	⊕⊕ **LOW**[6]	0.06 (-0.05, 0.18)

[1] Studies were considered at risk for imprecision if they did not meet the optimal information size criteria (<400 cases, 200 per group), or if the optimal information size is met, but the 95% CI includes 0.
[2] Included data from 12 randomized controlled trials, with a duration of follow-up ranging from 6 months to 7.7±1.8 years, enrolling participants from 4 different countries.
[3] Study quality (assessed by the Cochrane Collaboration's tool for assessing risk of bias) ranged from poor to good. 50% of studies included in this analysis were of poor quality, 33% were fair, and 17% were good.
[4] Optimal information size was met, however overall 95% CI of the mean difference crosses 0.
[5] Visual inspection of the funnel plot and Begg's test (p=0.061) suggests publication bias, whereas the Egger's test (p=0.525) does not.
[6] Data from randomized controlled trials begin with a grade of "HIGH". Downgraded due to risk of bias, imprecision, and publication bias.

	Stratified by dosage								
	<0.625 mg E-only HRT	589 (3)[7]	Not serious[8]	Not serious (I^2=0%)	Not serious	Serious[9]	Not assessed[10]	⊕⊕⊕ **MODERATE**[11]	-0.01 (-0.29, 0.28)
	≥0.625 mg E-only HRT	1118 (2)[12]	Not serious[13]	Not serious (I^2=37%)	Not serious	Serious[14]	Not assessed[15]	⊕⊕⊕ **MODERATE**[16]	-0.07 (-0.42, 0.27)
	<0.625 mg E + any P dose	570 (1)[17]	Not serious[18]	Not serious (I^2=0%)	Not serious	Serious[19]	Not assessed[20]	⊕⊕⊕ **MODERATE**[21]	-0.06 (-0.30, 0.19)
	≥0.625 mg E + any P dose	1553 (10)[22]	Not serious[23]	Not serious (I^2=0%)	Not serious	Serious[24]	Serious[25]	⊕⊕ **LOW**[26]	0.19 (-0.01, 0.39)

[7] Included data from 3 randomized controlled trials, with a duration of follow-up ranging from 2-3 years, enrolling participants from 2 different countries.
[8] Study quality ranged from poor to good. Thorneycroft et al. is a larger, good quality study with more participants contributing to this meta-analysis. Kenny et al. is a poor quality study with placebo women receiving progesterone as well. While ideally the control group would receive no hormones, due to the poor quality of this study, it is unlikely to impact the results.
[9] Optimal information size was met, however 95% CI of the mean difference crosses 0.
[10] Due to a small number of studies (n<10), publication bias was not formally assessed.
[11] Data from randomized controlled trials begin with a grade of "HIGH". Downgraded due to imprecision.
[12] Included data from 2 randomized controlled trials, with a duration of follow-up ranging from 2-6 years, enrolling participants from the USA.
[13] Study quality ranged from fair to good.
[14] Optimal information size was met, however 95% CI of the mean difference crosses 0.
[15] Due to a small number of studies (n<10), publication bias was not formally assessed.
[16] Data from randomized controlled trials begin with a grade of "HIGH". Downgraded due to imprecision.
[17] Included data from 1 randomized controlled trials, with a duration of follow-up of 2 years, enrolling participants from the USA.
[18] Study quality was good.
[19] Optimal information size was met, however 95% CI of the mean difference crosses 0.
[20] Due to a small number of studies (n<10), publication bias was not formally assessed.
[21] Data from randomized controlled trials begin with a grade of "HIGH". Downgraded due to imprecision.
[22] Included data from 10 randomized controlled trials, with a duration of follow-up ranging from 6 months to 6 years, enrolling participants from 4 different countries.
[23] Study quality ranged from poor to good. 60% of studies included in this analysis were of fair/good quality.
[24] Optimal information size was met, however 95% CI of the mean difference crosses 0.
[25] Visual inspection of the funnel plot suggests publication bias.
[26] Data from randomized controlled trials begin with a grade of "HIGH". Downgraded due to imprecision and publication bias.

©Javed AA et al. *JAMA Network Open*.

	Stratified by follow-up length								
	Shorter length	1644 (6)[27]	Not serious[28]	Not serious (I^2=0%)	Not serious	Serious[29]	Not assessed[30]	⊕⊕⊕ **MODERATE**[31]	0.00 (-0.16, 0.16)
	Longer length	2759 (4)[32]	Serious[33]	Not serious (I^2=0%)	Not serious	Serious[34]	Not assessed[35]	⊕⊕ **LOW**[36]	0.10 (-0.06, 0.27)

[27] Included data from 6 randomized controlled trials, with a duration of follow-up ranging from 6 months to 2 years, enrolling participants from 4 different countries
[28] Study quality ranged from poor to good. 66% were fair or good, 33% were poor.
[29] Optimal information size was met, however overall 95% CI of the mean difference crosses 0.
[30] Due to a small number of studies (n<10), publication bias was not formally assessed.
[31] Data from randomized controlled trials begin with a grade of “HIGH”. Downgraded due to imprecision.
[32] Included data from 4 randomized controlled trials, with a duration of follow-up ranging from 2.9 ±1.1-6 years, enrolling participants from 2 different countries.
[33] Study quality ranged from poor to fair. 75% were of poor quality.
[34] Optimal information size was met, however overall 95% CI of the mean difference crosses 0.
[35] Due to a small number of studies (n<10), publication bias was not formally assessed.
[36] Data from randomized controlled trials begin with a grade of “HIGH”. Downgraded due to risk of bias and imprecision.

©Javed AA et al. *JAMA Network Open*.

	Stratified by time since menopause								
	Shorter	2092 (5)[37]	Serious[38]	Not serious (I^2=0%)	Not serious	Serious[39]	Not assessed[40]	⊕⊕ **LOW**[41]	0.01 (-0.13, 0.16)
	Longer	1989 (3)[42]	Serious[43]	Not serious (I^2=26%)	Not serious	Serious[44]	Not assessed[45]	⊕⊕ **LOW**[46]	0.16 (-0.10, 0.42)

[37] Included data from 5 randomized controlled trials, with a duration of follow-up ranging from 6 months to 5 years, enrolling participants from 4 different countries.
[38] Study quality ranged from poor to good. 80% were of poor quality.
[39] Optimal information size was met, however 95% CI of the mean difference crosses 0.
[40] Due to a small number of studies (n<10), publication bias was not formally assessed.
[41] Data from randomized controlled trials begin with a grade of "HIGH". Downgraded due to risk of bias and imprecision.
[42] Included data from 3 randomized controlled trials, with a duration of follow-up upto 6 years, enrolling participants from 2 different countries.
[43] Study quality ranged from poor to fair. Although 67% were of fair quality, this was downgraded due to poorly reported information.
[44] Optimal information size was met, however 95% CI of the mean difference crosses 0.
[45] Due to a small number of studies (n<10), publication bias was not formally assessed.
[46] Data from randomized controlled trials begin with a grade of "HIGH". Downgraded due to risk of bias and imprecision.

©Javed AA et al. *JAMA Network Open*.

	Stratified by study quality								
	Fair/Good	3449 (6)[47]	Not serious	Not serious (I^2=0%)	Not serious	Serious[48]	Not assessed[49]	⊕⊕⊕ **MODERATE**[50]	0.04 (-0.09, 0.17)
	Poor	952 (6)[51]	Serious	Not serious (I^2=0%)	Not serious	Serious[52]	Not assessed[53]	⊕⊕ **LOW**[54]	0.20 (-0.08, 0.48)

[47] Included data from 6 randomized controlled trials, with a duration of follow-up ranging from 6 months to 6 years, enrolling participants from 4 different countries.
[48] Optimal information size was met, however 95% CI of the mean difference crosses 0.
[49] Due to a small number of studies (n<10), publication bias was not formally assessed.
[50] Data from randomized controlled trials begin with a grade of "HIGH". Downgraded due to inconsistency and imprecision.
[51] Included data from 6 randomized controlled trials, with a duration of follow-up ranging from 6 months to 5 years, enrolling participants from 4 different countries.
[52] Optimal information size was met, however 95% CI of the mean difference crosses 0.
[53] Due to a small number of studies (n<10), publication bias was not formally assessed.
[54] Data from randomized controlled trials begin with a grade of "HIGH". Downgraded due to risk of bias, inconsistency, and imprecision.

©Javed AA et al. *JAMA Network Open*.

	Stratified by type of muscle measurement								
	DEXA	4095 (7)[55]	Not serious[56]	Not serious (I^2=0%)	Not serious	Serious[57]	Not assessed[58]	⊕⊕⊕ **MODERATE**[59]	0.06 (-0.06, 0.18)
	Other	271 (4)[60]	Serious[61]	Not serious (I^2=0%)	Not serious	Serious[62]	Not assessed[63]	⊕⊕ **LOW**[64]	0.07 (-0.43, 0.57)

[55] Included data from 7 randomized controlled trials, with a duration of follow-up ranging from 6 months to 6 years, enrolling participants from 3 different countries.
[56] Study quality ranged from poor to good. 57% were of fair/good quality.
[57] Optimal information size was met, however 95% CI of the mean difference crosses 0.
[58] Due to a small number of studies (n<10), publication bias was not formally assessed.
[59] Data from randomized controlled trials begin with a grade of "HIGH". Downgraded due to imprecision.
[60] Included data from 4 randomized controlled trials, with a duration of follow-up ranging from 12 months to 3 years, enrolling participants from 3 different countries.
[61] Study quality ranged from poor to good. 50% were of poor quality.
[62] Optimal information size was met, however 95% CI of the mean difference crosses 0.
[63] Due to a small number of studies (n<10), publication bias was not formally assessed.
[64] Data from randomized controlled trials begin with a grade of "HIGH". Downgraded due to risk of bias and imprecision.

©Javed AA et al. *JAMA Network Open*.

eFigure. Funnel Plot for Assessment of Publication Bias

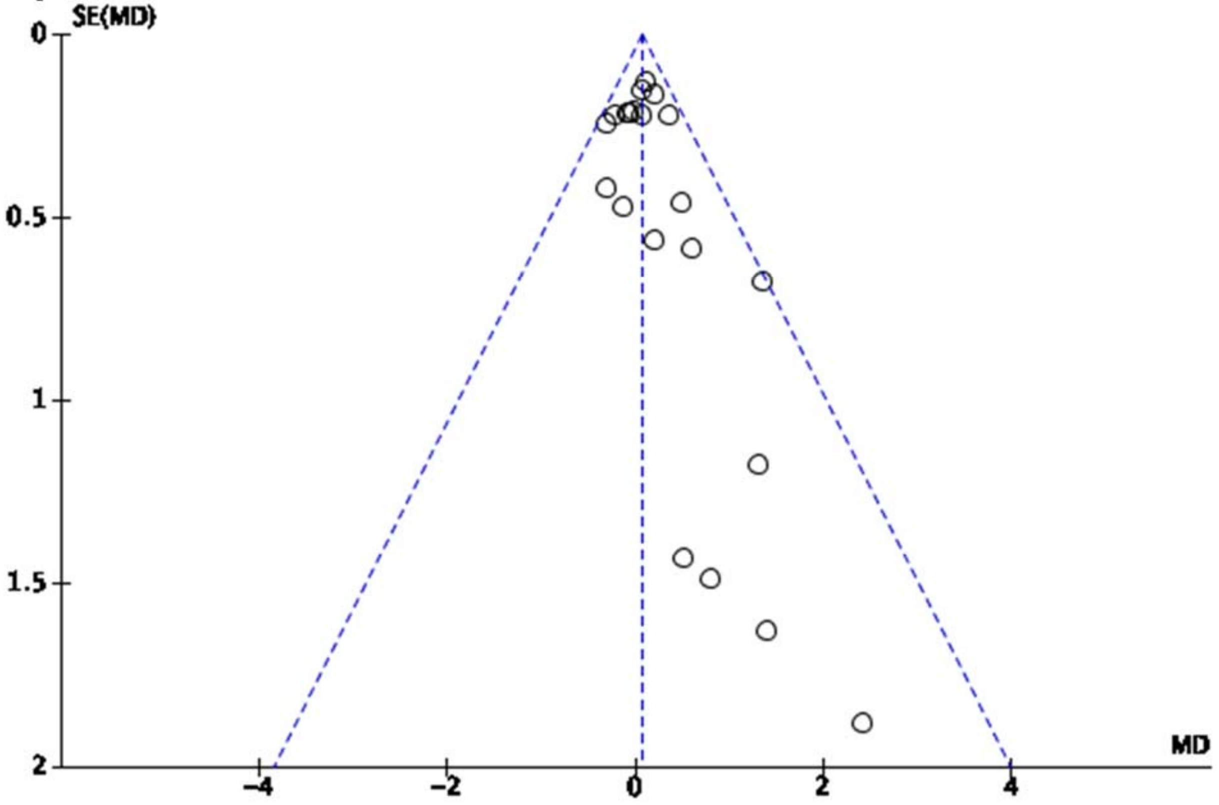

Caption: A visual inspection of the funnel plot of effect size and precision presents some asymmetry, indicating potential publication bias.

©Javed AA et al. *JAMA Network Open*.
